# Prenatal nicotine exposure leads to epigenetic alterations in peripheral nervous system signaling genes in the testis of the rat

**DOI:** 10.1186/s13072-024-00539-5

**Published:** 2024-05-07

**Authors:** Ouzna Dali, Jose Antonio Muriel-Muriel, Ana Vargas-Baco, Sergei Tevosian, Jasenka Zubcevic, Fatima Smagulova, Linda F. Hayward

**Affiliations:** 1 EHESP, Inserm, Irset (Institut de recherche en sante, environnement et travail)-UMR_S 1085, Univ. Rennes, 35000 Rennes, France; 2 Department of Physiological Sciences, University of Florida, 1333 Center Drive, Box 100144, Gainesville, FL 32610 USA; 3 Department of Physiology and Pharmacology, University of Toledo, Toledo, OH USA

**Keywords:** Nicotine, Testis, Pituitary gland, Histone marks, MeDIP, RNA-seq

## Abstract

**Background:**

Prenatal nicotine exposure (PNE) has been documented to cause numerous deleterious effects on fetal development. However, the epigenetic changes promoted by nicotine exposure on germ cells are still not well understood.

**Objectives:**

In this study, we focused on elucidating the impact of prenatal nicotine exposure on regulatory epigenetic mechanisms important for germ cell development.

**Methods:**

Sprague-Dawley rats were exposed to nicotine during pregnancy and male progeny was analyzed at 11 weeks of age. Testis morphology was analyzed using frozen testis sections and expression of germ cell markers was examined by RT-qPCR; histone modifications were assessed by Western Blot (WB). DNA methylation analysis was performed by methylation-specific PCR of bisulfite converted DNA. Genome-wide DNA methylation was analyzed using Methylated DNA immunoprecipitation (MeDIP)-seq. We also carried out transcriptomics analysis of pituitary glands by RNA-seq.

**Results:**

We show that gestational exposure to nicotine reduces germ cell numbers, perturbs meiosis, affects the expression of germ line reprogramming responsive genes, and impacts the DNA methylation of nervous system genes in the testis. PNE also causes perturbation of gene expression in the pituitary gland of the brain.

**Conclusions:**

Our data demonstrate that PNE leads to perturbation of male spermatogenesis, and the observed effects are associated with changes of peripheral nervous system signaling pathways. Alterations in the expression of genes associated with diverse biological activities such as cell migration, cell adhesion and GABA signaling in the pituitary gland underscore the complexity of the effects of nicotine exposure during pregnancy.

**Supplementary Information:**

The online version contains supplementary material available at 10.1186/s13072-024-00539-5.

## Background

Smoking, and the indirect passive nicotine exposure, remains a global public concern. Despite known risks, many pregnant women struggle to quit nicotine and continue to smoke during pregnancy. According to a study published in 2018 by Lancet Global Health, the rate of smoking during pregnancy in Europe is among the highest in the world with ~ 8% of pregnant women affected [1]. Epidemiological studies provide an unequivocal connection between smoking during pregnancy and health issues in children. Maternal smoking is thought to contribute to reduced fetal growth, preterm birth, abnormal brain development, respiratory and cardiovascular issues and a higher risk of obesity, diabetes, hypertension, and other health problems later in life of the child [2–4]. Nicotine exposure is linked to obesity and thyroid malfunction [5–7], perturbed nervous system development [8], as well as behavioral impairment such as Attention-Deficit/Hyperactivity Disorder (ADHD) [9, 10]. Importantly, nicotine exposure was documented to affect the development of the male reproductive system, where it perturbs sperm motility and production of sex hormones [11].

This study is part of a comprehensive project which was aimed to assess whether nicotine exposure (that occurs in humans as a result of consumption of either electronic or standard cigarettes) during pregnancy affects the gut microbiota in the rat, and how alterations in microbiota composition impact development of the nervous, cardiovascular, digestive, and reproductive systems [12]. This work was initiated by Dr. Linda Hayward at the University of Florida. Hayward and colleagues demonstrated that gut microbiota affects general acetylation levels by increasing concentration of acetylated groups in the blood [4]. This study is aimed to reveal the epigenetic effects of nicotine on the reproductive system in male offspring.

Spermatogenesis is a process culminating in the formation of male haploid gametes. The quality of gametes is important and extends beyond solely fitness to conceive, as it is critical to the development of healthy progeny. Thus, understanding the consequences of PNE on germ cells is important for developing policies and regulations to mitigate the risks associated with nicotine exposure in women. The first germ cell population forms during embryonic development from somatic cells. During development, global reprogramming of the embryo occurs in which the entire genome undergoes rounds of demethylation and re-methylation to establish new cellular layers. After two waves of reprogramming, the first population of germ cells emerges. Germline responsive genes (GRRs) play a key role during germ cell formation. This group of 45 genes is controlled by the ten-eleven translocation (TET) proteins; GRRs gradually lose their methylation marks while their expression increases [13, 14]. A majority of GRRs are meiotic-specific genes. It has been shown that perturbation of demethylation at these genes results in a reduction of the germ cell population [13]. Thus, the epigenetic state of GRRs is critical, and perturbations in their methylation and expression can lead not only to a decrease in germ cell numbers, but also to meiosis failure.

Spermatogenesis resumes after birth in testis seminiferous tubules, a network of tubular structures composed from the periphery to the lumen of less differentiated to most differentiated cellular layer. Gonocytes ranging from immature to fully developed include spermatogonia, spermatocytes, round spermatids, elongated spermatids, and spermatozoa that reside in the tubules. In addition to gonocytes, Sertoli cells are large and tightly connected support cells of the testis that facilitate the spermatogenesis process directly or indirectly through hormonal negative feedback systems. The testis also contains star-shaped Leydig cells, residing between seminiferous tubules, that produce sex hormones. The somatic cells of the testis assist spermatogonia to undergo replication, and those tetraploid cells subsequently enter meiosis, to give rise to haploid gametes.

The initiation of meiosis requires the coordinated action of intrinsic and extrinsic factors. An important feature of meiosis is crossing overs, homologous recombinations allowing DNA exchanges between pairs of homologous chromosomes. This process is required for proper chromosome segregation before the first meiotic division. Epigenetic mechanisms are thought to be intimately involved in the regulation of meiosis and spermatogenesis. Specifically, histone modifications such as γH2AX [15], H3K9me3 [16], H3K4me3 and H3K36me3 [17], and ubiquitination of histones H2A [18] and H2B [19] play key roles in meiotic progression.

Here, we hypothesized that nicotine exposure occurring during pregnancy could alter the epigenetic landscape of the whole organism in utero. Gestational exposure could promote epigenetic alterations in germ cell reprogramming and these changes could persist in male progeny after birth.

We show that gestational exposure to nicotine reduces germ cell numbers, perturbs meiosis, affects the expression of germline reprogramming responsive genes, and impacts DNA methylation at nervous system genes in the testis that regulate hormonal signaling. In addition, several genes related to cell migration, cell adhesion and GABA signaling are affected in the pituitary gland of nicotine-exposed animals.

## Results

### Gestational nicotine exposure alters testis morphology and decreases germ cell numbers

**Fig. 1:**
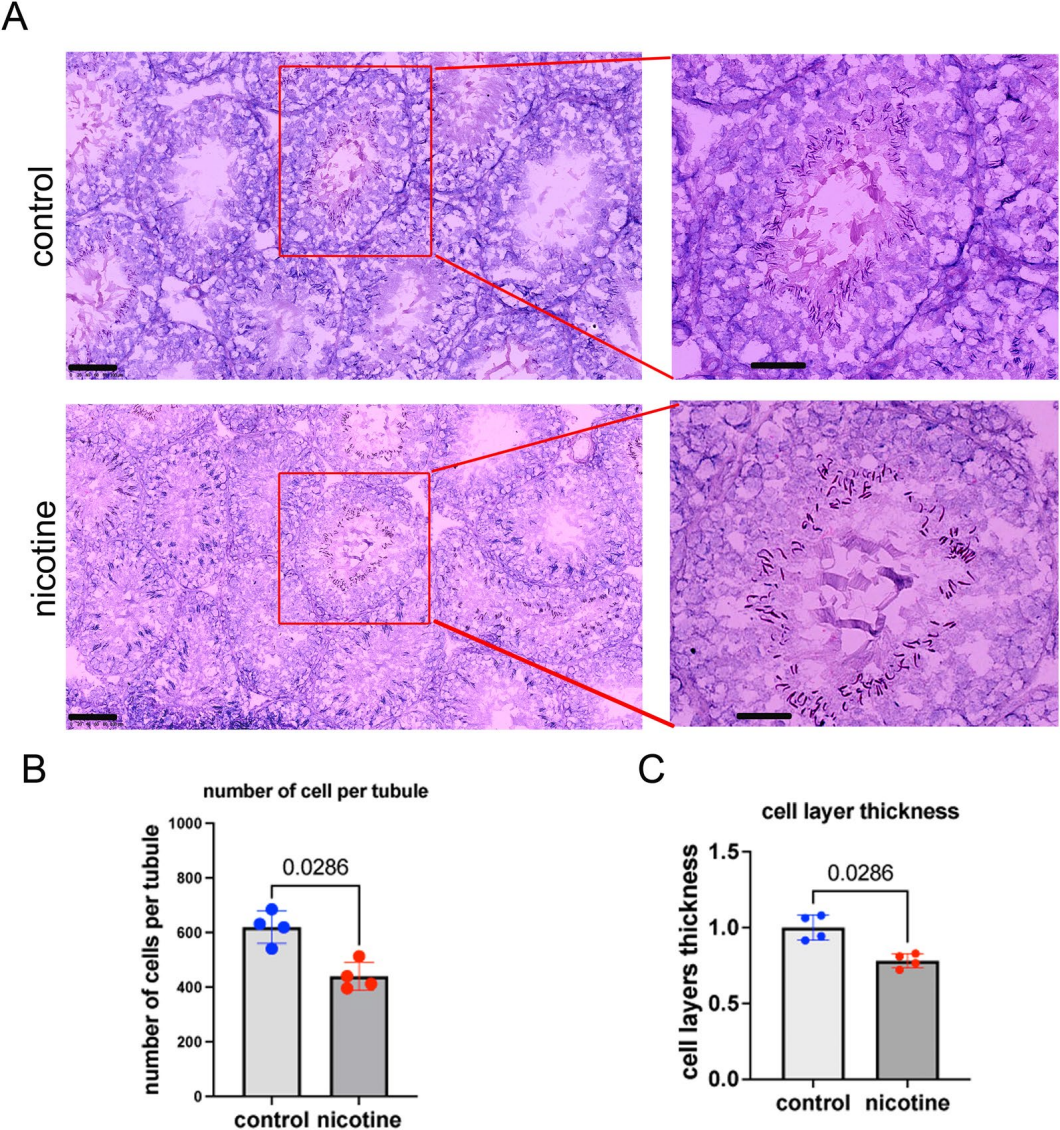
Testis morphology in Sprague-Dawley male rats prenatally exposed to nicotine. **A** Representative images of testes from mice exposed in utero to 0 (top) and 6 mg/kg/day (bottom) of nicotine. Sections were stained with H&E, scale bars are 100 µM (left panels) and 50 µM (right panels). **B** Number of cells per tubule. **C** Thickness of seminiferous tubules, n = 4 dose 0 mg/kg/day and n = 4 dose 6 mg/kg/day; significance, exact p-value are indicated on the top of the graph, the p -values were determined by the Mann-Whitney nonparametric test. Rats were sacrificed at 11 weeks of age. The cells were counted in stage (VI-VII) tubules. All plots in the Figure represent averaged values ± standard deviation

To explore the effects of maternal nicotine exposure on testis morphology of the progeny, we compared hematoxylin and eosin-stained frozen testis sections in 11-week-old male animals born to nicotine-exposed or control mothers. We examined the tubules at stage VI-VII in which all cell types are present to focus on the homogenous presence of the cell types between groups. In rats, there are 14 stages of spermatogenesis that can be distinguished in seminiferous tubules [20]. The various stages are represented by different types and numbers of cells [20]. The spermatogonia and early meiotic cells (leptotene-zygotene stage) are normally found in proximity to the membrane of seminiferous tubule cells, whereas more advanced pachytene and haploid cells are closer to the lumen (Fig. 1A). Stages VI and VII in the rat testis are defined based on the appearance and development of specific germ cells within the seminiferous tubules. Stage VI is characterized by the presence of late spermatids with elongated heads. The acrosome and nuclear shaping are more pronounced, and these spermatids are approaching the final stages of their maturation process. One of the defining events of Stage VII is the release of mature spermatids from the seminiferous epithelium into the lumen of the tubules. This stage is marked by the presence of the most mature spermatids at the luminal edge of the epithelium. Furthermore, primary spermatocytes at the preleptotene/leptotene stage, which are just about to enter meiosis, are typically seen. Thus, we decided to choose these stages to avoid the high diversity of cell density derived from the difference in stages of spermatogenesis. We analyzed frozen testis sections stained with hematoxylin and eosin. The images were generated using nanoZoomer, and cells in stage VI-VII seminiferous tubules were counted. The number of cells localized in seminiferous tubules were divided by the area. We counted cells in a minimum of 6 tubules from 4 different control and 4 nicotine-exposed testis. We found that the cell density decreases by 0.7-fold (p = 0.03, nonparametric Mann-Whitney test) as a result of nicotine exposure (Fig. 1B).

We also examined the thickness of the cell layer. We used the Fiji tool [21] and measured the parameter “perimeter”. We drew a line from the outer membrane of the seminiferous tubule to its center and measured the distance to the lumen. We compared the average values from at least 20 tubules from 4 different controls and 4 nicotine-exposed testes. We found a 0.78-fold (p = 0.03, nonparametric test) decrease in seminiferous tubule thickness at stage VI-VII in exposed animals compared to controls (Fig. 1C).

**Fig. 2:**
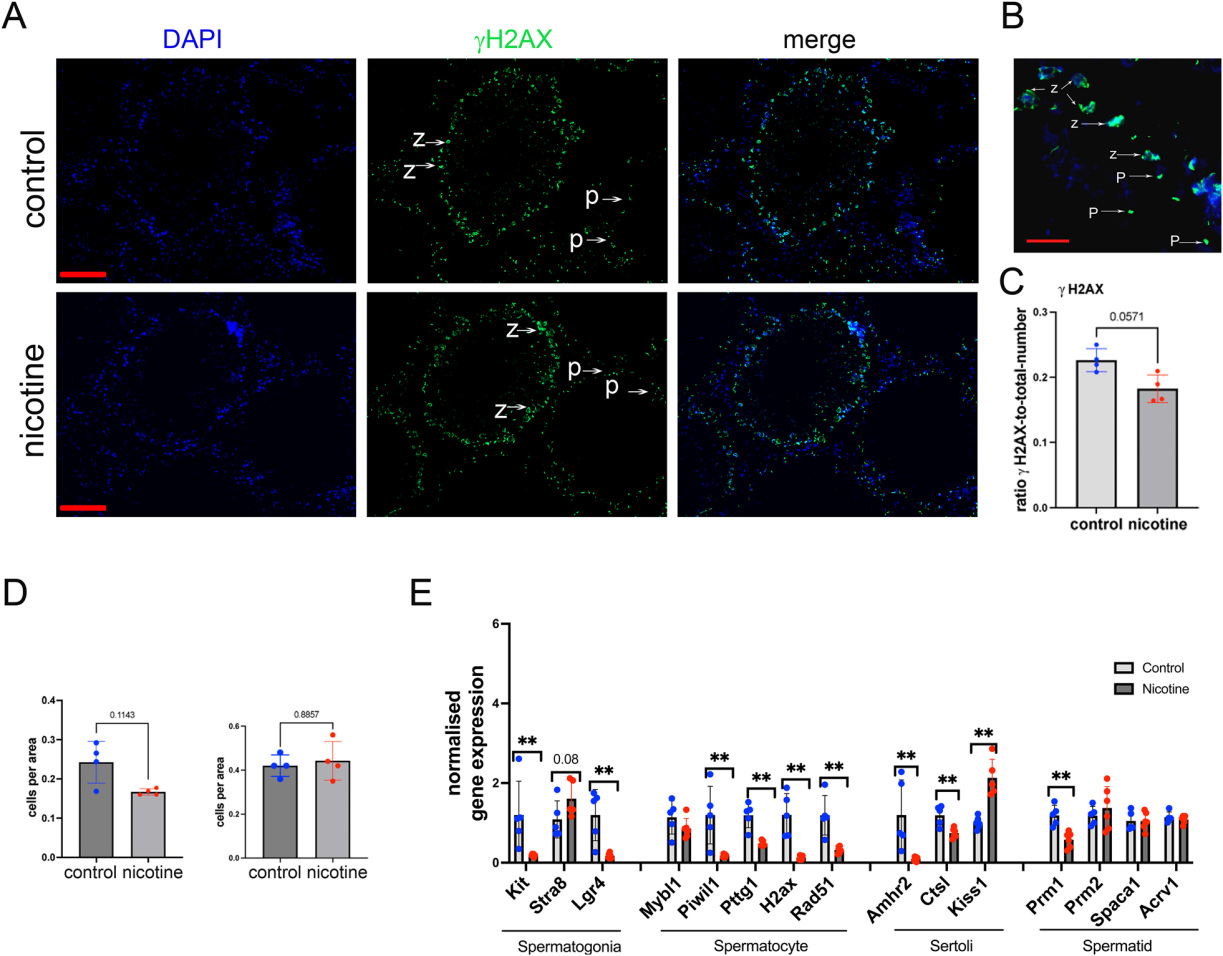
The effects of gestational nicotine exposure on meiotic and somatic testis cell. **A** Representative images of 11 week-old male testis spreads immunostained for γH2AX (green) in control (top) and nicotine (bottom) exposed testis; scale bar is 50 µM. **B** High magnification image (63X objective), z-leptotene-zygotene cells, p-pachytene-diplotene cells, scale bar is 20 µM. **C** Quantitative analysis of the ratio of γH2AX-positive to total number of cells; n = 4, dose 0, n = 5 dose 6 6 mg/kg/day, **p < 0.01 or p-value is indicated on the top of the graph, nonparametric Mann-Whitney test. **D** Quantitative analysis of leptotene -zygotene (left) or pachytene-diplotene (right) γH2AX-positive cells, p-value is indicated on the top of the graph, nonparametric Mann–Whitney test. **E** Analysis of gene expression in testis of 11 week-old rats by RT-qPCR; n = 5 dose 0, n = 6 dose 6 mg/kg/day. All plots represent average values ± standard deviation

Next, we analyzed the number of germinal meiotic cells using the γH2AX marker. The non-canonical phosphorylated histone variant H2AX replaces the canonical histone H2A during formation of double-strand breaks (DSBs), thus allowing the chromosomes to become more condensed (reviewed in [22]). DSBs formation occurs throughout the whole genome and γH2AX marks are widely spread at each chromosome. At early stages of meiosis (leptotene-zygotene) when DSBs start to form, γH2AX shows a very bright staining all over the nucleus. At the later stage (pachytene-diplotene), when autosomes segregate and DSBs are getting repaired, γH2AX marks are retained only at sex-chromosomes as a bright spots [23]. γH2AX staining at cells such as Sertoli cells or type 2 spermatocytes, spermatogonia and spermatids is normally weak and generally only appears as foci corresponding to occurrence of random DNA damage. We performed γH2AX analysis on frozen testis sections. The sections were fixed with 4% PBS-buffered paraformaldehyde, washed and blocked, and the immunofluorescence assay was performed using an antibody against γH2AX (Fig. 2A, B) as described in the Methods section. The images were taken with objective 20 × and fixed time of exposure. We scored γH2AX positive cells (green staining) and compared them to the total number of cells in the tubules (DAPI staining). Our analysis revealed that nicotine exposure decreased the number of yH2AX-positive cells but the reduction was not statistically significant (0.8-fold decrease of γH2AX-positive cells, p = 0.06, nonparametric Mann-Whitney test, Fig. 2C). We also counted cells with a γH2AX pattern specific of leptotene-zygotene (bright staining all over the nucleus) and cells with a pattern specific of pachytene-diplotene (small bright spot in the nucleus). We found that nicotine exposure caused a non-significant decrease in the number of leptotene-zygotene-positive cells (p = 0.11, nonparametric Mann-Whitney test, Fig. 2D, left) and did not alter the number of pachytene-diplotene-positive cells (Fig. 2D, right). Thus, a small decrease in germ cell number, likely due to reductions in leptotene-zygotene and other cell types such as spermatogonia, could contribute to the decrease in total germ cell numbers even though the decrease in the number of leptotene-zygotene cells did not reach statistical significance.

To reveal which cell populations might be impacted by nicotine, we analyzed the expression of genes which are specifically expressed in each cell population based on a recently published dataset [24]. To this end, we extracted total RNA from whole testis and performed quantitative RT-qPCR analysis (Fig. 2E). Our analysis revealed that nicotine exposure decreased the expression of genes that are specifically expressed in spermatogonia, *Kit* and *Lgr4*, by 0.16- (p = 0.004), and 0.15-fold (p = 0.008), respectively. In contrast, the expression of *Stra8* tended to increase 1.5-fold (p = 0.1). We also determined that nicotine exposure decreased the expression of several genes normally expressed in spermatocytes, *Piwil1, Pttg1, H2ax* and *Rad51*, by 0.15 (p = 0.004), 0.4 (p = 0.004), 0.1 (p = 0.004), and 0.27-folds (p = 0.004), respectively. The *Kiss1* gene that is specific for Sertoli cells was increased by 2.13-fold (p = 0.004) by nicotine exposure; in contrast, *Ctsl and Amhr2* genes which are also expressed in Sertoli cells were decreased by 0.6 (p = 0.008) and 0.08-folds (p = 0.004), respectively (Fig. 2E). Finally, *Prm1,* specific for the spermatid fraction was decreased by 0.48 (p = 0.004) by nicotine exposure (Fig. 2E). The most dramatic changes in gene expression were in the spermatogonia and spermatocyte fractions. Considering the relatively small decrease in germ cell numbers, we believe that these changes in gene expression reflect a decrease in these cell types.

In conclusion, the morphology analysis demonstrated that nicotine exposure causes a small but significant decrease in the germ cell population, with a tendency toward decrease of the leptotene-zygotene cells. We also observed perturbations in the expression of genes specific for each cell type, suggesting that prenatal exposure to nicotine alters gene expression in almost all cell types of the testis. However, the changes in expression of spermatogonia and spermatocytes genes could be due to a decrease in these cell populations.

### Nicotine exposure perturbs meiotic progression

**Fig. 3:**
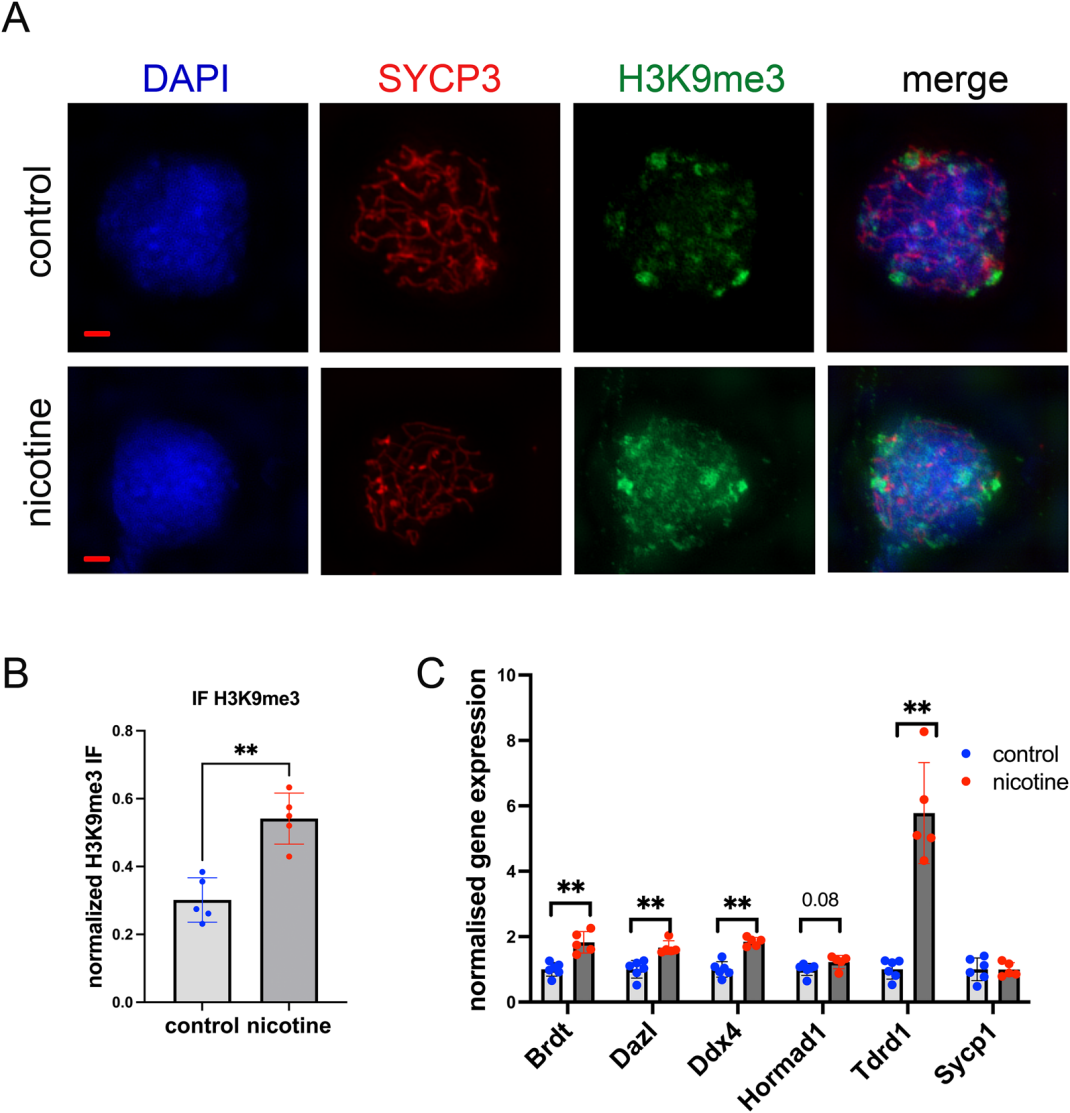
The effect of gestational nicotine exposure on H3K9me3 levels and GRRs gene expression. **A** Representative images of 11 week-old male testis spreads immunostained for H3K9me3 (green) or SYCP3 (red, to visualize chromosome) in control (top) and nicotine-exposed mice; scale bar is 5 µM. **B** Quantitative analysis of H3K9me3 staining intensity; n = 5 dose 0, n = 6 dose 6 mg/kg/day, **p < 0.01, nonparametric Mann-Whitney test. **C** Quantitative analysis of GRRs gene expression in testis of 11 week-old males, n = 6 dose 0, n = 5 dose 6 mg/kg/day, **p < 0.01 or p-value indicated on the top of column, nonparametric Mann-Whitney test. All plots represent average values ± standard deviation

To further explore effects of nicotine exposure on meiosis we analyzed the formation of H3K9me3 marks in nuclei. During meiosis H3K9me3 marks are normally localized all over the nucleus in the leptotene-zygotene stages and become concentrated at the nuclear periphery at the pachytene-diplotene stages [25]. H3K9me3 is normally enriched at regions of compact heterochromatin during meiosis (reviewed in [26]). Normal meiosis is characterized by patches of H3K9me3 marks mainly at the nuclear periphery, and these can be perturbed in certain pathological conditions in which alteration of telomere attachment and reduction of H3K9me3, leads to a loss of chromatin repression at telomeres [27]. To analyze H3K9me3 marks, cells were dissociated from whole testis and passed through a cell strainer to make cell spreads on slides. The spreads were fixed with paraformaldehyde, washed, blocked and immunostained for H3K9me3 (Fig. 3A) and SYCP3, a chromosome marker. Z-stack images were taken with similar exposure time and a quantitative analysis of the mark’s intensity was performed using imageJ as described in Methods. H3K9me3 and DAPI intensity were measured, and the H3K9me3 signal was normalized to DAPI staining. The analysis was performed for a minimum of 10 cells for each biological replicate (4 controls and 4 nicotine exposed), the averaged values were compared and plotted and the nonparametric Mann-Whitney test was used. We found a 1.7-fold increase in H3K9me3 staining in PNE cells, suggesting a possible impact of nicotine on normal chromosome segregation (Fig. 3B).

Next, we sought to analyze the expression of meiotic GRR genes given their importance for optimal meiosis. Our analysis showed that most of the tested GRR genes were increased by nicotine exposure: *Brdt, Dazl, Ddx4, Tdrd1* displayed a 1.5-, 1.6-, 1.7-, and 5.0-fold increase, respectively, suggesting a potential perturbation of reprograming at GRR loci by nicotine exposure (Fig. 3C). *Hormad1* expression which is essential for meiosis [28] also showed a trend towards increase by 1.2-fold (Fig. 3C).

In summary, meiosis and GRRs gene expression were perturbed in PNE male rats suggesting that PNE has a deleterious effect on the reproductive system in adults.

### The H3K9me3 regulatory histone marks are increased in nicotine exposed testis

**Fig. 4:**
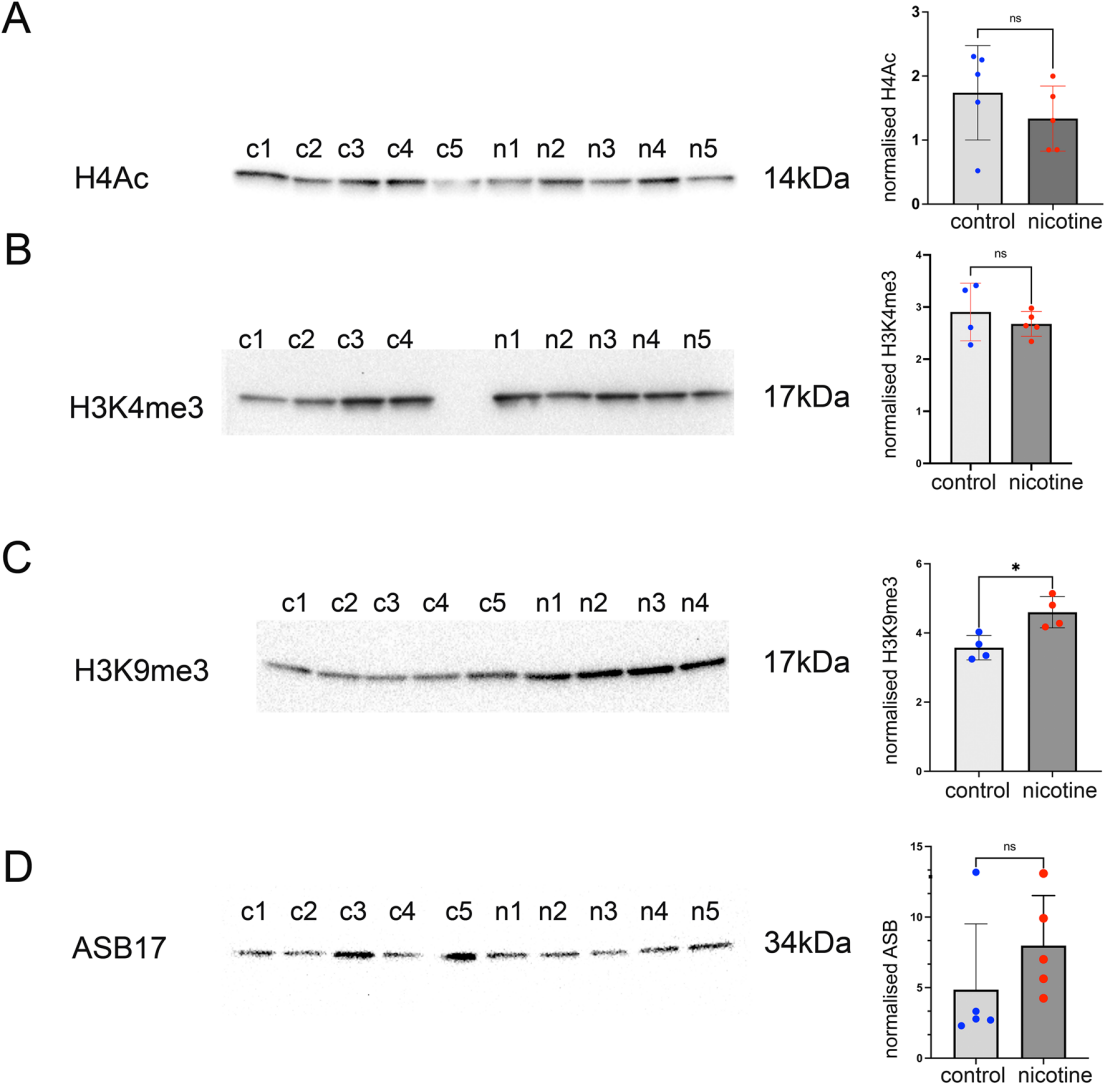
Analysis of histone marks and expression of the spermatid marker ASB17 in testis of control and nicotine-exposed rats. **A** Representative images of H4Ac, **B** H3K4me3, **C** H3K9me3, and **D** ASB17. c1-c5 are controls and n1-n5 are nicotine-exposed samples, *p < 0.05 nonparametric Mann-Whitney test

Since nicotine exposure leads to changes in microbiota and to an increase in short acid chain groups known to increase acetylation of histones [12], we decided to analyze major regulatory histone levels which are important during meiosis [29]. The changes in histone acetylation could perturb other histone marks as histone acetylation and histone methylation often compete for same lysine residues. For instance, acetylation and methylation of a given lysine site are mutually exclusive, thus the methylation at that site can prevent activation by acetylation [30]. To check the level of modified histones in testis more precisely, we purified the histones from whole testis as described in the Methods section and performed the quantitative analysis of modified histones (Additional file 3: Fig S1). The signal of histone marks was normalized to Ponceau red staining (Additional file 3: Fig S2). First, we analyzed presence of acetylated histone H4. We did not observe a significant change in H4Ac levels in nicotine-exposed testis (Fig. 4A). We acknowledge here that the analysis was performed on adult testis while the exposure occurred during the embryonic development, hence we may be observing compensatory effects.

Next, we analyzed the presence of H3K4me3 (which marks open chromatin). Our analysis showed no significant changes in the global level of H3K4me3 in response to nicotine exposure, although we cannot exclude the possibility of regional changes (Fig. 4B).

We also analyzed H3K9me3 mark which are associated with gene silencing and are abundant at compact heterochromatin. Here, in contrast, H3K9me3 marks were significantly increased in nicotine-exposed samples (Fig. 4C). The results of H3K9me3 marks detected by WB are consistent with the immunofluorescence results (Fig. 3A, B), which also demonstrated higher level of H3K9me3 in spermatocytes suggesting a global impact of nicotine exposure on H3K9me3.

To determine the effect of nicotine on haploid cell populations we analyzed the level of the ASB17 protein by WB (Fig. 4D). *Asb17* is expressed exclusively in the testis, and *Asb17* deficiency leads to a significant reduction of apoptosis in germ cells [31]. The highest expression of *Asb17* is detected in round spermatids [32], suggesting its role in spermiogenesis. To analyze its expression, we extracted proteins from the testis and performed analysis by WB. We found that ASB17 levels were not significantly changed in nicotine-exposed cells (Fig. 4D), suggesting that the number of haploid cells is not notably perturbed, and changes in cell population are due to alterations of less mature cell types such as spermatocytes. We also performed the analysis of PRM2 levels. We found that PRM2 levels were not significantly changed in nicotine-exposed cells (Additional file 3: Fig S3).

In summary, higher level of H3K9me3 during meiosis caused by nicotine exposure can affect compact heterochromatin during meiosis. WB analyses suggest the absence of global changes in post-meiotic haploid cells, indicative of no developmental delay occurring post-meiosis.

### Nicotine affects DNA methylation in the peripheral nervous system signaling and transcription factor genes

**Fig. 5:**
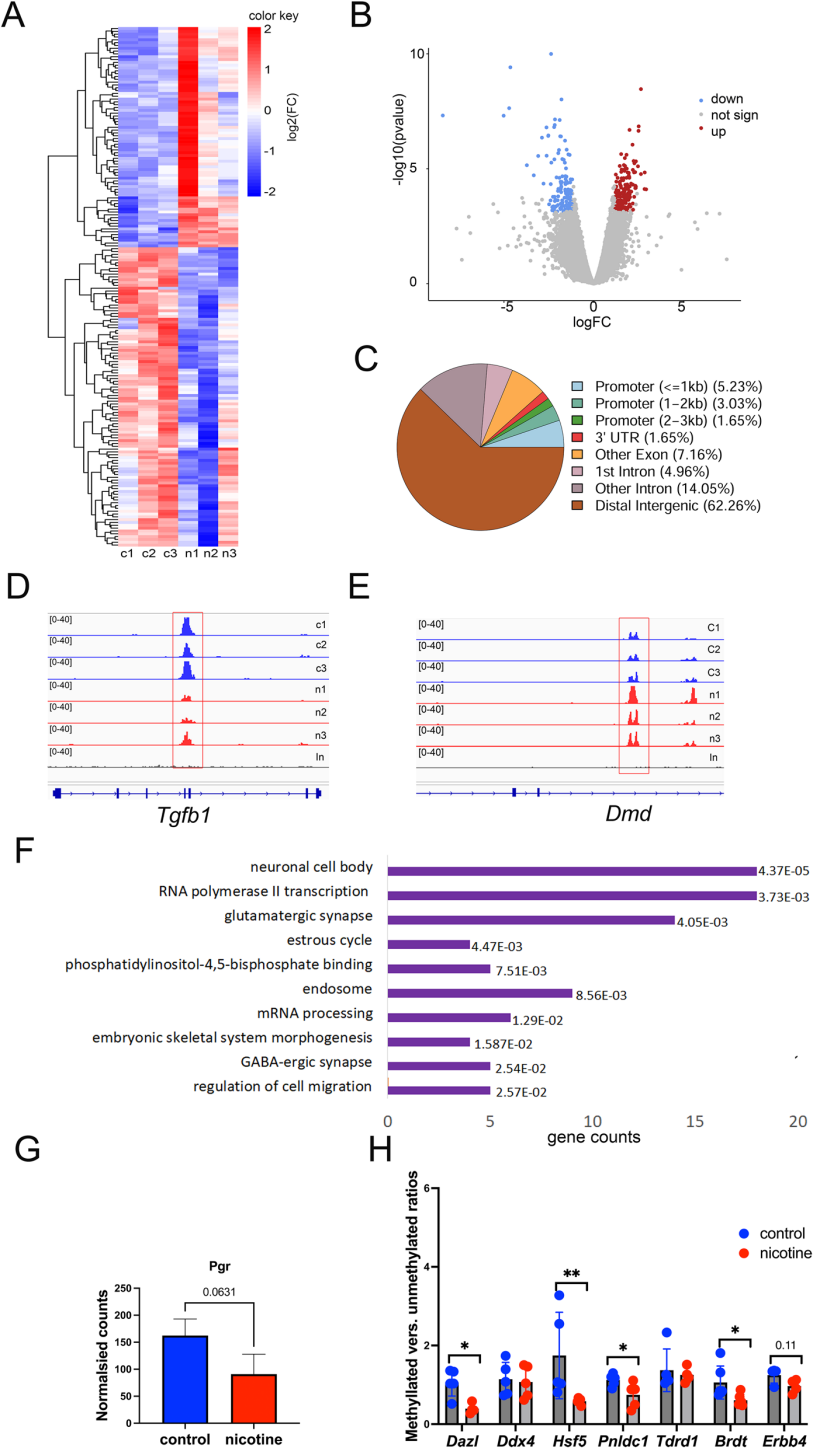
Genome-wide DNA methylation analysis of testis from 11-week-old rats. **A** Heatmap of all annotated DMRs. Counts of genes with FC > 1.5 and FDR < 0.1 were log transformed and plotted in R using Pheatmap, and as input log2 counts of DMRs regions were used. **B** Volcano plot of DMRs. **C** Genomic localization of DMRs determined by ChIPseeker. The differentially methylated peaks within *Tgfb1* (**D**) and *Dmd* **(E)** are shown in red dashed boxes. Plots from control (*blue*) and nicotine-exposed (*red*) samples are shown. Each control and treatment group contained three replicates. The sequencing reads were mapped to the reference rn7 *Rattus norvegicus* genome, normalized and converted to bedgraph files, which were visualized in IGV. The signal intensity is shown in brackets. **F** Functional annotation as “biological process”, “cellular localization” or “molecular function’ of genes located in common DMRs by DAVID. Bars were sorted by adjusted p-values and the length of each bar represents the number of genes in this group. **G** Normalized DNA methylation at the vicinity of *Pgr* gene, counts were extracted from sequencing data and plotted in Excel, exact p-value are indicated on top of columns. **H** Methylation -specific PCR. DNA from testis was bisulphite-converted and used for qPCR using primers specific for methylated and unmethylated DNA. ΔΔCq Method was used for the analysis of the ratio of methylated-to-unmethylated DNA. The average ratios were plotted, *p < 0.05. **p < 0.01, or exact p-value is indicated on top of the column, statistical significance was estimated by the nonparametric Mann-Whitney test

To explore the impact of nicotine on DNA methylation we performed DNA methylation analysis using a genome-wide sequencing technique. Briefly, we extracted DNA form testis and carried out DNA methylation enrichment experiments using the EpiMark enrichment kit (NEB). The kit is based on the purification of methylated DNA fragments bound to the methyl-CpG binding domain of human MBD2 protein, which is fused to the Fc tail of human IgG1, and coupled to protein A beads. After several wash steps, the enriched DNA sample is eluted from the beads and used for library construction. The library was built by using NEBNext^®^ Ultra DNA Library Prep Kit (Illumina) using dual labeled multiplexed primers. The sequencing was performed by the Genomics platform, and obtained reads were filtered and aligned to the rat reference rn7 genome. The number of reads was normalized by size factors. The regions with peaks were determined using both methylated enriched and input libraries. The reads were counted at the peaks for each biological replicate and statistical test was performed. Nearly ~ 57,000 of methylated regions were detected. We identified 366 regions that showed differential pattern of DNA methylation (FDR < 0.1) in response to nicotine exposure (Fig. 5A, Additional file 1). Among differential peaks, 198 showed increased methylation and 165 regions showed decreased DNA methylation (Fig. 5B, Additional file 3). Analysis using the ChIPseeker package showed that most of the differentially methylated regions (DMRs) are genomic elements located distally from the gene promoters. However, nearly ~ 14% of DMRs were found in introns (Fig. 5C). To determine differentially methylated genes based on the DMRs, we assigned DMRs to genes using ChIPseeker. Functional annotations of DMRs by DAVID showed that they are enriched in genes that are expressed in neuronal cell body (*Dmd, Tgfb1)* (Fig. 5D, E); genes encoding transcription factors (*Foxj3, Nfact2, Rfx3, Meis3, Rbpj, Foxs1, Dmtf1, Hoxb3, Hoxa2, Tead1, E2f7, Hoxa5, Hoxb5, Creb5*), the glutamatergic synapse proteins (*Qsec2, Dtnbp1, Cabp1, Begain, Dnm2, Olfm1, Actc1, Slitrk2, Psd2, Prkar2a, Slitrk1, Syt10, Gpc6, Adgrl3*), and GABAergic signaling factors (*Slitrk2, Slitrk1, Sst, Iqsec3, Igsf9b*) (Fig. 5F). In addition, the genes encoding estrous cycle-related factors (*Trpm2, Anxa1, Cyp1b1, Pgr*) harbored DMRs (Fig. 5F).

Notably, nicotine exposure caused a decrease in DNA methylation of the progesterone receptor gene (Fig. 5G). Testicular progesterone is a by-product of steroidogenesis which is not converted into testosterone. Serum 17-OHP appears to be a reliable proxy marker for intratesticular testosterone levels and could potentially be used as a readout to titrate or change medications that alter intratesticular testosterone. Based on this data we believe that nicotine exposure likely perturbs testosterone levels [33].

To confirm the DNA methylation state of GRRs by methylation-specific PCR, we performed bisulfite conversion of testis DNA and designed methylation-specific PCR primers. We used Methprimer (http://www.urogene.org/methprimer/index.html [34]) to design primers for methylated and unmethylated bisulfite-converted DNA PCR and checked the specificity of PCR products (Additional file 3: Fig S4A–D) by analyzing the melting curves. We compared the methylated-to-unmethylated ratios using the ΔΔCq method, and statistical significance of methylated-to-unmethylated PCR ratio was determined using the nonparametric Mann–Whitney test. This analysis confirmed that GRRs in nicotine-exposed mice have a lower DNA methylation level (e.g., *Dazl, Hsf5, Phdlc1, Brdt*) (Fig. 5H). These changes negatively correlate with gene expression, suggesting the strong impact of nicotine on GRRs.

**Fig. 6:**
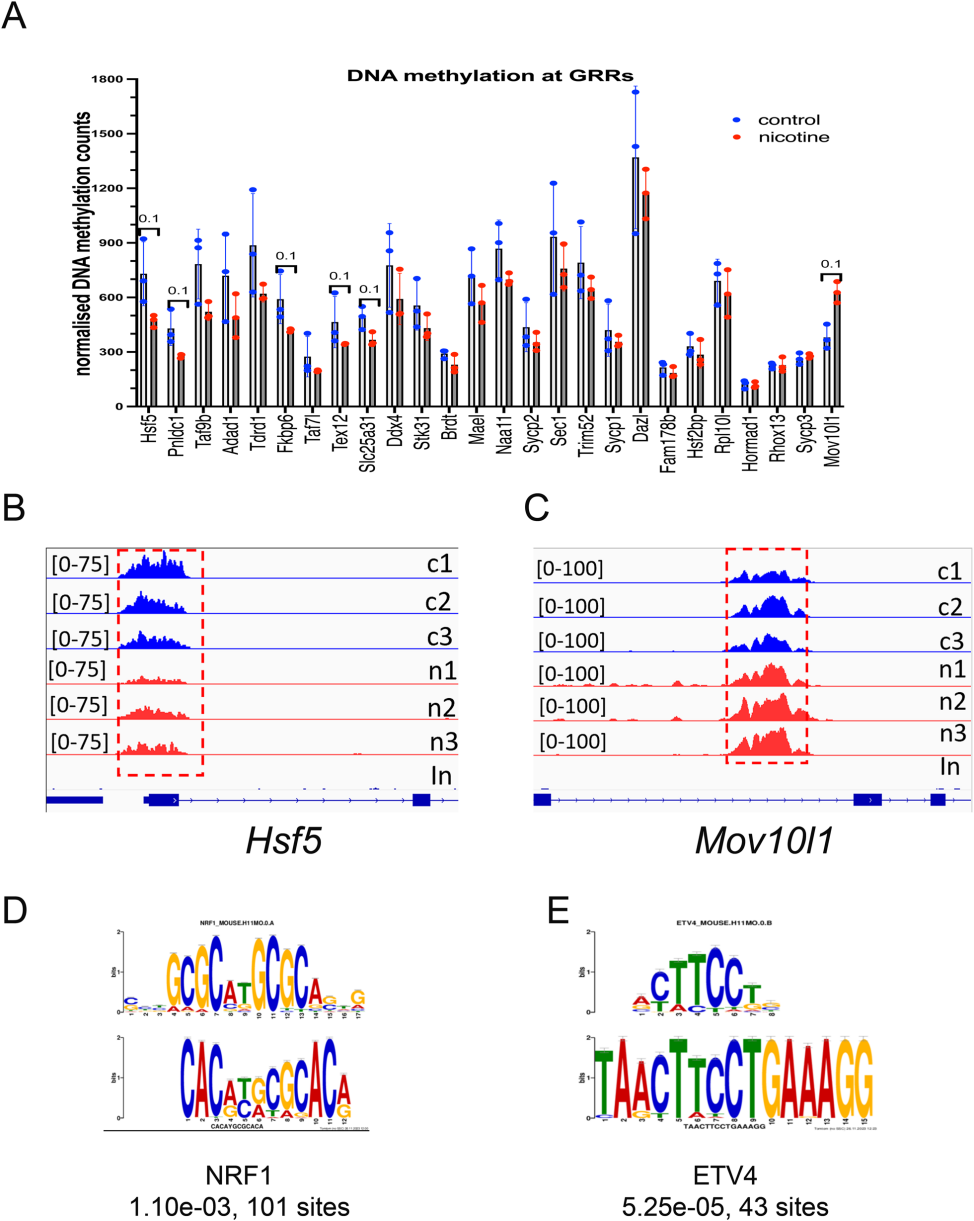
DNA methylation analysis of GRRs. **A** Normalized DNA methylation of GRRs. Counts were extracted from sequencing data and plotted in Excel, exact p-value are indicated on top of columns (nonparametric Mann–Whitney test, FDR test was not applied). The differentially methylated peaks near **B** *Hsf5* and **C** *Mov10l1* are shown in red dashed boxes. Plots from control samples are shown in blue, plots from nicotine-exposed samples are shown in red. Each control and treatment groups contained three replicates. The signal intensity is shown in brackets. **D** Motif analysis by MEME-ChIP revealed two enriched motifs, parts of these motifs are significantly similar to NRF1 and ETV4 binding sites

Due to their importance for meiosis, we specifically examined the DNA methylation status of 45 GRRs. We extracted the sequencing read numbers of the methylated regions of GRRs and plotted them in Excel (Fig. 6A, Additional file 3:S1). We found that DNA methylation marks are present at ~ 30 genes. We observed that DNA methylation has a tendency to decrease at the *Hsf5 (p* = *0.1,* nonparametric test) and is increased at the *Mov10l1* gene (p = 0.1, nonparametric test) in response to nicotine exposure (Fig. 6B). Notably, decreased DNA methylation at GRRs negatively correlates with GRRs gene expression (Fig. 3C) suggesting a possible impact of PNE on DNA methylation at GRRs during development and on expression of these genes in adulthood.

We also searched for the presence of motif enrichments at DMRs to identify a possible mechanism of action of PNE. To this end, we extracted sequence information of DMRs in *fasta* format and performed a motif search using MEME-ChIP which performs comprehensive motif analyses (including motif discovery) on sequences in which motif sites tend to be centrally located, such as ChIP-seq peaks. We used the eukaryotic motif database MOUSE/HOCOMOCOv11 for the search. Our analysis determined that two common motifs are found in many sequences. Part of the first common motif is similar to the binding site for the NRF1 transcription factor (Fig. 6D). *Nrf1* encodes a regulator of mitochondrial metabolism that plays a critical role in the development of post-migrating primordial germ cells (PGC) [35], suggesting the role of this factor in nicotine-treated germ cells. The conditional ablation of NRF1 in gonocytes dramatically down-regulates several germline genes (*Dazl, Lin28a, Ddx4*), blocks germ cell proliferation, and subsequently leads to male infertility in mice [36] suggesting an important role of NRF1 in regulation of some GRRs.

Part of the second identified motif is similar to the binding site for ETV4 (Fig. 6E). ETV4 regulates cell proliferation [37]. *Etv4* mutant mice fail to form specific motor neurons, which do not branch normally within their target muscles, and the cell bodies of neurons are displaced within the spinal cord [38]. Thus, it is possible that the disfunction in ETV4 binding caused by nicotine could affect the formation and functioning of the peripheral nervous system in many organs including the testis. In this respect, while *Etv4*(–/–) males show normal mating behavior, they do not leave copulatory plugs and sperm is not detected in the uteri of females that mate with *Etv4*(–/–) males [39].

In summary, DNA methylation analysis uncovered that upon nicotine exposure there is differential methylation of genes relevant to nervous system signaling and transcription factor activity. We propose that abnormal activities of the NRF1 and ETV4 transcription factors could alter the development and proliferation of post-migratory primordial germ cells.

### RNA-seq analysis of the pituitary gland from nicotine-exposed animals reveals effects on genes important for cell migration, cell adhesion and GABAergic signaling.

**Fig. 7:**
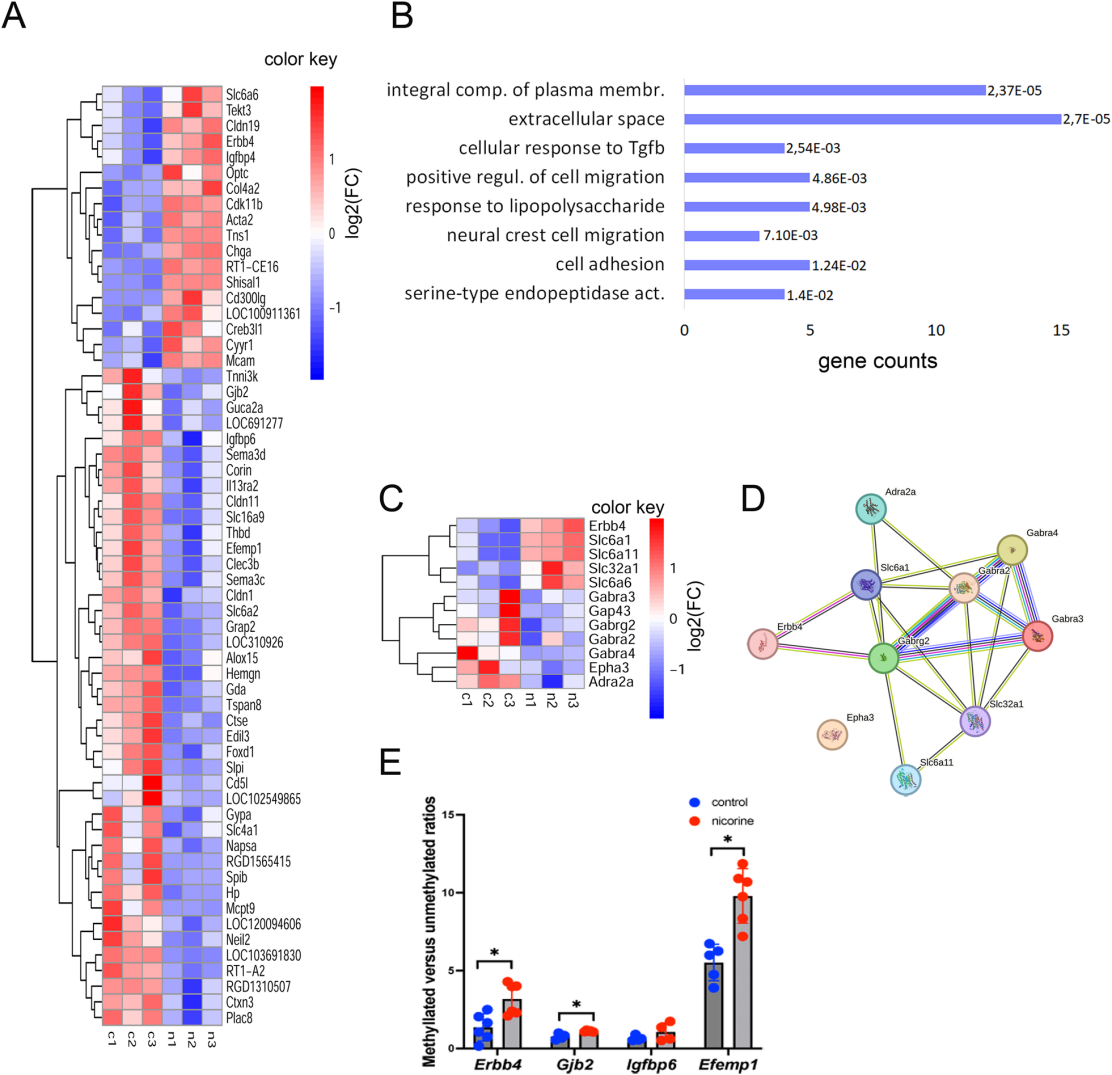
RNA-seq analysis in the pituitary gland. **A** Heatmap of all annotated DMRs. Normalised transcript per million counts (TPM) of genes (FDR < 0.1) were log transformed and plotted in R, c1-c3 control, n1-n3 nicotine exposed samples, upregulated genes are in red, downregulated genes in blue. **B** Functional annotation “biological process”, “cellular component” or “molecular function” of DEG genes according to DAVID, bars sorted by p-values, and each bar represents the number of genes in each group, the analysis was done without multiple testing correction. **C** Heatmap of GABAergic signalling genes. The TPMs were log transformed and plotted in R. **D** The network of GABAergic signalling genes, red line—indicates the presence of fusion evidence, green line—neighbourhood evidence, blue line—cooccurrence evidence, purple line—experimental evidence, black line—coexpression evidence, network was built by String (https://string-db.org/cgi/). **E** Methylation-specific PCR in the pituitary gland. DNA from pituitary glands was bisulphite-converted and used for qPCR using primers specific for methylated and unmethylated DNA. The ΔΔCq method was used for analysis of the ratio of methylated-to-unmethylated DNA. The averaged ratios were plotted, *p < 0.05, statistical significance was estimated by nonparametric Mann-Whitney test

To explore the molecular mechanisms of nicotine exposure in the neuroendocrine system, we performed transcriptomic analysis of the pituitary gland using paired-end stranded RNA sequencing using 3 biological replicates from the *nicotine*-treated and control rats. We chose to study the pituitary gland due to its key role for hormonal regulation of reproductive functions. Total RNA was extracted, treated with DNAse I, and strand-specific libraries were prepared and sequenced in multiplexed mode. Reads were mapped to the reference rn7 genome. The differentially expressed genes were determined (Additional file 2). The samples showed some variations between replicates (Additional file 3: Fig S5A, B), possibly due to the complexity of cellular milieu in the pituitary. However, we were able to identify 60 differentially expressed genes (DEGs) between nicotine exposed samples and controls (FDR < 0.1) (Fig. 7A, Additional file 3: Fig S5C, Additional file 3: Table S1). The majority of DEGs were downregulated in nicotine treatment samples (Fig. 7A, Additional file 3: Fig S5D). We performed a functional annotation of the DEGs with the gene ontology program DAVID. The strongest enrichment in DEGs was found in genes related to *Tgfb1* signaling, regulation of cell migration, and cell adhesion among others (Fig. 7B). It is noteworthy, that *Tgfb1* regulated genes (*Acta2, Col4a2)* were upregulated by nicotine exposure in the pituitary gland, in contrast to the decrease in *Tfgb1* DNA methylation observed in the testis of exposed animal, suggesting a possible link between pituitary and testis *Tfgb1* signaling pathways*.*

Since we observed some alteration in DNA methylation of GABAergic signaling genes in the testis, we decided to take a closer look at these genes in the pituitary gland RNA-seq data. To this end, we catalogued genes that are combined by the gene ontology term “GABAergic synapse” using the AMIGO database (https://amigo.geneontology.org/amigo). 136 genes were found related to this term, with 121 out of them expressed in the rat pituitary gland. We determined that *Erbb4* and *Slc6a6* were significantly upregulated upon nicotine exposure and three other genes (*Slc6a11, Slc32a1, Slc6a6*) showed a tendency to increase. In contrast, six other genes (*Gabra4, Gabrg2, Epha3, Gabra3, Gap43, Adra2a*) showed a tendency to decrease (Fig. 7C). These altered genes encode for proteins that are combined in network with central players GABRA2, GABRA3, GABRA4 and GABRG2 (Fig. 7D).

Similar to our approach with testis GRRs genes’ analysis, we decided to explore DNA methylation in some DEGs using methylation-specific PCR. We extracted pituitary gland DNA and performed the bisulfite conversion. Primers for methylation specific PCR were designed and qPCR analysis was carried out. Our results demonstrated that nicotine exposure is associated with increased DNA methylation in intron 1 of *Erbb4* gene (Fig. 7E), within the CpG region located in the gene body. Importantly, methylated CpG islands in the gene body positively correlate with gene expression [40]. We also found an increase of DNA methylation in the promotors of *Gjb2* and *Efemp1* genes which negatively correlates with gene expression (Fig. 7E). We chose to analyze these genes due to their importance for the brain function. Mutations in *Gjb2* gene are responsible for the recessive deafness [41, 42]. Moreover, both nicotine and smoke exposure are strongly associated with the risks for developing sensorineural hearing loss [43]. Study showed that EFEMP1 is a paracrine activator of Notch signaling in endothelial cells and promotes glioma angiogenesis [44]. The increase in DNA methylation of these genes is negatively correlate with the gene expression, suggesting that that nicotine exposure during embryonic development could perturb the epigenetic regulation of these genes.

In summary, RNA-seq analysis of the pituitary showed that expression of genes related to *Tgfb1* signaling, cell migration, cell adhesion and GABAergic signaling was altered between the control and the nicotine-exposed group, suggesting that PNE affects the neuroendocrine system as well. DNA methylation of several DEGs was similarly altered suggesting that gestational exposure to nicotine could also induces changes in the pattern of DNA methylation in the pituitary.

## Discussion

In this work, we examined testes of male offspring from rats exposed to nicotine during pregnancy. Our analysis showed that testis morphology in PNE animals was changed. Specifically, we observed in reduction in the total number of germ cells per tubule. This could possibly be explained by the effects of nicotine exposure on GRR genes as they are known to impact the formation of the entire population of germ cells [13]. We conclude that nicotine exposure has the potential to globally affect meiosis. We also observed gene expression changes in germ line (spermatogonia and spermatocytes) as well as in somatic (Sertoli) cells suggesting that nicotine could perturb meiosis by acting on both the somatic and germ cell compartment. For example, a decrease in the expression of *Rad51* (recombinase) could lead to alterations in homologous recombination.

While we cannot exclude the possibility that nicotine directly induces the changes we observed in the testis, we believe an indirect effect to be more plausible. Changes in gene methylation in the nervous system could affect the production of sexual hormones, and the observed effects could be due to perturbation of nervous system signaling. Specifically, we observed alterations in DNA methylation of the molecular components of the glutamatergic system (Fig. 5F). Glutamate transporters (VGLUTs) are expressed in the testis; for example, VGLUT2 is expressed and localized to the outer acrosomal membrane of spermatids, suggesting that the glutamatergic system has a function in the testis [45]. In this respect, GABA, a major neurotransmitter in the central nervous system, also acts as a signaling molecule in endocrine tissues such as the pituitary and testis ([46]; reviewed in [47]). Testicular Leydig cells are both producers and targets of GABA signaling [48]. These cells express GABA (A) receptor subunits, and pharmacological activation of the murine Leydig cell line TM3 with GABA or GABA (A) agonists leads to increased proliferation [49–51].

Importantly, we found an increase in the expression of the *Erbb4* gene in the pituitary gland of nicotine-exposed rats. Notably, ERBB4 have been linked to nicotine addiction as genome-wide association (GWA) and candidate gene studies for smoking behavior and nicotine dependence revealed that ERBB4 is a strong candidate for nicotine dependence, and several mutations were identified in addicted individuals [52]. On the other hand, it has been reported that nicotine controls synaptic plasticity through NRG3/ErbB4-dependent regulation of GABAergic inhibition [53]. A significant increase in *Erbb4* and *Nrg3* expression were reported following chronic nicotine exposure and withdrawal in mice [52]. Thus, it is possible that nicotine consumption increases the expression of *Erbb4* which in turn exerts an inhibitory effect on GABAergic signaling. The small decrease in GABAergic receptor genes in our study is in line with this hypothesis.

We also observed a decrease of the DNA methylation of GABAergic signaling genes such as *Iqsec3, Igsf9b* and *Sst* in the testis. Notably, *Sst* encodes for the somatostatin protein. In testis the SST receptor is expressed in Leydig cells [54], and somatostatin is known to inhibit proliferation of male germ cells [55]. Thus, decreased levels of *Sst* methylation could affect its expression and impact germ cell proliferation. We also suggest that, similar to the pituitary tissue, the GABAergic effects on the testis could be additionally mediated via *Erbb4*, as ERBB4 is expressed in Sertoli cells and affects Leydig cells through paracrine signaling [56]. Further work is required to confirm this mechanism.

Western blots and DNA methylation analyses suggest that the overall repression of the genes studied caused by nicotine exposure is mediated by a reduction of open chromatin complexes and an increase in repressive H3K9me3 histone mark. As DNA methylation and histone marks are functionally linked, it is conceivable that alterations in either DNA methylation or histone marks could induce a cascade of events leading to a global change in chromatin architecture and gene expression.

Furthermore, we observe an alteration of DNA methylation at important germline reprogramming genes. Pilsner et al. evaluated the influence of cigarette smoking on sperm-epigenetic aging (SEA). They suggested that SEA could be estimated by measuring time-to-pregnancy (TTP) and they showed by DNA methylation analysis of sperm samples that higher SEA is associated with longer TTP. SEA was higher among males who were current cigarette smokers [57]. In addition, besides fertility problems, we believe that epigenetic alterations in sperm could contribute to the aberrant development of organ systems (reviewed in [58]). Ashapkin and colleagues report that there is growing evidence that an altered epigenome and phenotype of the offspring are linked with the accumulation of epigenetic changes over time in the father's sperm. Chemical and lifestyle exposures may contribute to epigenetic aging of sperm [58].

## Conclusion

In summary, we determined that PNE affects the expression of a wide range of genes related to transcription and nervous system function. We suggest that alterations in gene expression and epigenetic control perturb germ cell proliferation and normal testis development. This study revealed several biomarker genes (*Erbb4, Tgfb1, Nrf1, Etv4, Efemp1, Gjb2*) that could be useful for the analysis of nicotine effects in human biological samples.

## Materials and methods

### Experimental design

Outbred Sprague-Dawley pregnant rats were treated either with vehicle (control) or a low nicotine dose. After treatment, the male offspring were euthanized at 11 weeks of age and testes were harvested. Specifically, we analyzed testis morphology, profiled gene expression and meiosis as well as documented histone status by Western blot. We also performed genome-wide analysis of DNA methylation in the testis (MeDIP-seq) and completed bioinformatic analysis to identify signaling pathways affected by gestational exposure to nicotine. Finally, we performed transcriptomic analysis of the pituitary gland using stranded paired RNA-sequencing to identify new biomarkers of exposure.

### Animal treatment

Specific pathogen-free male and female Sprague Dawley rats were purchased from Envigo (USA). To reduce the number of animals used in the study, the same animals were used in for experiments described in this study and for microbiome analyses for a separate study. Specific pathogen free (SPF) animals are routinely used in such studies to maintain controlled baseline microbiota, reduce confounding variables, and enhance reproducibility as well as improve animal health. Females were purchased at 8 weeks of age and were allowed 1 week to acclimate. At 9 weeks of age, females were randomly assigned to one of 4 treatment groups. Two groups of females were mated, and two groups of females remained virgins. Mating included placing a single female overnight with a male and examination on the following morning for the presence of sperm via vaginal swabs. If sperm was present, the dam was weighed and placed in a separate cage, and this period was marked as gestational day (GD) 0. If no sperm was detected, the pair remained together, and the female was examined daily until pregnancy and GD0 were confirmed. Using a sterile technique, a 28-day osmotic mini-pump (2ML4-ALZET, Durect Corp., Cupertino, CA, USA) filled with either sterile saline (control; CON) or a nicotine tartrate (6 mg/kg; NIC), dissolved in sterile saline was placed subcutaneously between the scapulae, as described previously [59, 60]. On GD19, or 13 days following implant of the pump, dams gave birth.

Male progeny from both control and nicotine-exposed dams was sacrificed at 11 weeks of age. Animals were anesthetized at a surgical plane with isoflurane (2.5% in 100% oxygen) for blood collection. Following this, all animals were given an overdose of sodium pentobarbital (> 200 mg/kg). Several organs, including pituitary and testes in the males, were collected and preserved in liquid nitrogen.

### RNA extraction, quantification & cDNA synthesis and RT-QPCR

Testicles stored at – 80 ºC from the control and treatment group were used for RNA extraction. Testis samples were lysed and homogenized using TissueLyser (Qiagen). Approximately 30 mg of testis tissue was used (6 biological replicates) for RNA extraction using RNeasy Plus Mini Kit 250 (Qiagen, 74134). Purified RNA was eluted in 50 µL of RNase-free water. RT was performed using 1 µg of total RNA with iScript (Bio-Rad, 1708891) adhering to the Minimum Information for Publication of Quantitative Real-Time PCR Experiments (MIQE) guidelines Bustin et al. (2009). We used *Rpl37a* as a housekeeping gene because it showed no variation between replicates based on RNA-seq data. Results are presented as the fold change relative to the control ± SD. Primers for this study were selected using the Primer-Blast program from ncbi.nih.gov, and most of them included exon-to-exon junctions; the primers are listed in Additional file 3:Table S2A. A nonparametric Mann-Whitney test was used for evaluating statistical significance.

### MeDIP, library preparation and MeDIP-seq analysis

DNA was extracted from 6 biological replicates using DNA Easy Blood and Tissue Kit (Qiagen, 69506) according to instructions provided by the manufacturer. DNA concentrations were measured using a fluorescent reader (Promega). 10 µg of DNA was used for MeDIP using methylated the DNA Enrichment Kit (NEB, #E2600S). DNA was diluted and sonicated in a Qsonica 700 sonicator (Q700-110, Newtown, Connecticut, USA) supplied with cup horn 431C2 using the following conditions: 20 s pulse on, 20 s pulse off, total sonication time 8 min; these parameters generated ~ 300 bp DNA fragments. Methylated DNA was then extracted with A/G beads and the concentration of methylated DNA was again determined by fluorescence. Equal amounts of methylated DNA and input (7 ng) were used for library preparation. Sequencing libraries were prepared with the NEBNext Ultra DNA Library Prep Kit for Illumina (E7645S; NEB). Sequencing was performed on an Illumina HiSeq4000 sequencer using a paired-end 50-base read in multiplexed mode. An average of 44 million sequencing reads per sample were processed. The exact number of reads per sample is provided in Additional file 3:Table S3. The reads were mapped to the reference genome rn7 v2.5.0 using Bowtie (Langmead et al. 2009). The numbers of mapped reads were normalized by a scale factor to adjust the total number of reads using the Samtools program. From the aligned reads, DNA methylated peaks were identified using six biological replicates and the corresponding input by the MACS2 (2.2.7.1) algorithm [61]; the following parameters were applied: a shift-size window of 73 bp, no model, and a q-value threshold < 0.05. To compare the nicotine-exposed and control samples, differential peaks were identified using counting reads at each peak using bedtools MultiCovBed (Version 2.30.0) [62]. Statistical significance was calculated using DeSeq2 (v2.11.40.8) [63] with filtering peaks with low counts. For a finding DMRs, we used 3 replicates of control and 2 from nicotine group. We performed functional annotation of the differential peaks using the web-based tool ChIPseeker v1.28.3 [64] (using default parameters). For the visualization of ChIP-seq tracks, each.bam file was converted to BedGraph tracks by using Genome Coverage 2.30.0. IGV was used to visualize the tracks. Functional annotation of genes localized in differentially methylated regions was performed with the DAVID program using “Biological process”, “Molecular function”, “Cellular Component” terms [65]. The sequencing data were uploaded to the Galaxy web platform, and we used the public server at usegalaxy.org to analyze the data [67].

### Methylation- specific PCR.

500 ng of genomic DNA from testis or pituitary gland was bisulfite-converted using EpiTect Fast Bisulfite Conversion Kits from Qiagen (59,802) according to the protocol provided by the manufacturer. The concentration of bisulfite converted DNA was estimated by Nanodrop (Thermo Scientific). DNA was diluted to a concentration of 1 ng/μl and 4 μl was used for PCR. Primers for methylated and unmethylated amplification are listed in Additional file 3: Table S2B. We used a specific program Methprimer http://www.urogene.org/methprimer/index.html [34] to design primers for methylated and unmethylated bisulfite-converted DNA PCR. The coordinates of the CpG region localized close to promoters were extracted using DNA methylation profile from testis bedgraph files (our data) and the sequences of the regions were obtained from UCSC https://hgdownload.soe.ucsc.edu/downloads.html using option “get DNA”, in which we specified “repeat masking” to avoid PCR amplification of repeated regions. The sequence was placed into the program Methprimer for primer design. We checked the specificity of each PCR product by analysing the melting curve. For example, the promoter of *Brdt* showed higher level of unmethylated DNA compared to methylated (Additional file 3: Fig S3A). In contrast, *Gjb2* which is expressed in pituitary gland showed higher level of DNA methylation in promoter compared to unmethylated (Additional file 3: Fig S3B). The melting curves showed a single peak for each product (Additional file 3: Fig S3C, D). The Cq value for each unmethylated and methylated sequence was used for ΔΔCq analysis. The ratio of methylated-to-unmethylated PCR was calculated for each sample and the averaged ratios were compared between control and nicotine groups. Statistical significance was estimated by nonparametric Mann-Whitney test (Additional files 1, 2).

### RNA sequencing and data processing.

We used three biological replicates from the control group and three for nicotine group of rats. RNA was extracted using a RNeasy kit as described above. RNA was additionally treated with DNAse I (QIAGEN, 79254). One microgram of total RNA was used for a strand-specific library preparation protocol using NEBNext Ultra II Directional RNA Library according to the protocol provided by the manufacturer. Quality control and genome-wide sequencing were performed at the GenomEast platform at the Institute of Genetic, Molecular and Cellular Biology (IGBMC), Strasbourg, France. The sequencing was performed as massive parallel sequencing using paired-end mode, and the size of the sequencing tag was 100 bp. Reads in FASTQ format were processed for quality control using the FastQC tool (http://www.bioinformatics.babraham.ac.uk/projects/fastqc/). An average of 61 million sequencing reads per sample were processed. The exact number of reads per sample is provided in Additional file 3: Table S4. The reads were mapped to the reference genome [Rattus Norvegicus rn7 sequence] using the HISAT2 [66] alignment program with default parameters, and the alignment files were generated as BAM files. These files were used as the input for FeatureCounts to calculate the gene abundance using the gene annotation file ncbiRefSeq.gtf.gz from UCSC. Differential gene expression was assessed using the package DESeq2 (2.11.40.7) with the option of filtering genes with low counts. Genes were considered as differentially expressed if FC > 1.5 and FDR < 0.1. Functional annotation of differentially expressed genes (DEGs) was performed with DAVID [65] program using “Biological process”, “Cellular component” or “Molecular function” ontologies as background set for the genes which are normally expressed in adult pituitary gland.

### Histone extraction

The protein extraction was conducted using a Histone Extraction Kit (Abcam, ab113476) according to the protocol provided by the manufacturer. Testes stored at – 80 ºC were used for histone extraction. Briefly, samples were homogenized with a TissueLyser (Qiagen) and cell extracts were pelleted via centrifugation. After centrifugation and resuspension, the supernatant with histone extracts was collected and 0.3 volumes of Balance-DTT Buffer were added. Histone protein concentrations were quantified using Bio-Rad iMark^™^ with a microplate reader and reading at 660 nm using Bradford solution.

### Protein extraction

Small piece of tissue (~ 5 mg) and 300 μL of ice-cold lysis RIPA buffer (50 mM Tris-HCl, pH 8.0, 150 mM sodium chloride, 1.0% Igepal CA-630 (NP-40), 0.5% sodium deoxycholate, 0.1% sodium dodecyl sulfate (SDS)) were added rapidly to the tube and the tissue was homogenized with a tissue lyser homogenizer (Qiagen) and metal beads. The beads were rinsed with another 300 μL of lysis buffer. The homogenized tissue was agitated for 2 h at 4 °C and centrifuged for 20 min at 12,000 rpm at 4 °C. Tubes were placed on ice; the supernatant was transferred to a fresh tube and kept; the pellet was discarded. The protein concentration was estimated with a Bradford assay. Serial dilutions of bovine serum albumin (BSA) solution were used as a protein standard. 5 µg of protein/lane was loaded on the gel.

### Western blotting

Western blots were performed for the control and nicotine exposed samples using the following antibodies: rabbit anti-H3K4me3 (1:10000, Millipore 07-473), rabbit anti-H3K9me3 (1:10000 Abcam, ab8898), rabbit anti-ASB17 (1:1000, Abcam, 19800), goat anti-protamine 2 (1:1000, Santa Cruz, sc23104). Extracted histones (as described above) or protein extract were used for Western blotting. Concentrations of each protein sample obtained from OD readings were used to determine an exact volume containing 10 µg of histone protein for each sample. Aliquots of 10 µL containing RIPA (50 mM Tris-HCl, pH 8.0, 150 mM sodium chloride, 1.0% Igepal CA-630 (NP-40), Laemmli 3X buffer, and sample volumes containing 10 µg of histone proteins were denatured at 95 ºC for 5 min and run on a 4–20% gradient acrylamide gel Mini-PROTEAN TGX Stain-Free Gels (BioRad). Proteins were transferred onto Polyvinylidene difluoride (PVDF) membranes using a Trans-Blot^®^ Turbo^™^ Transfer System (BioRad). Ponceau Red staining was conducted to determine the relative abundance of total histone proteins. Images were collected using Molecular Imager ChemiDoc^™^ XRS + with Image Lab^™^ Software (BioRad). Blocking was conducted using a 5% milk in 1X TBS Tween 0.05%. The primary antibodies were diluted to different ratios in 10 mL of the blocking solution, and incubated with the membrane overnight at 4 ºC. After three 5 min washes with 1X PBS Tween 0,05%, each membrane was incubated for 1 h in 10 mL of the blocking solution containing the corresponding secondary antibodies (1:10000 for anti-rabbit and 1:5000 for anti-mouse). After three 5 min washes with 1X PBS Tween 0,05% the blots were exposed with ECL Prime Western Blotting Detection Reagent (RPN2232; Amersham). Specific protein expression for each antibody was documented using a molecular imager. Ponceau Red staining was used to determine the relative abundance of total histone proteins. The intensities of total histone abundances were used to normalize the specific epigenetic markers. Intensity of the bands were measured with ImageJ-Fiji software. Western blotting signals were presented as averaged normalized values relative to Ponceau Red.

### RT-qPCR

Reverse transcription of 1000 ngs RNA was performed with the iScript cDNA synthesis (BioRad). Reactions were loaded into 384-Well PCR plates (BioRad) along with iTaq Universal SYBR^®^Green Supermix (BioRad), primers (Eurogentec), and MilliQ water. Each well contained 5 μL of SYBR^®^Green Supermix, 0,05 μL of each primer pair of primers (100 µM stock solution), 0,9 μL of H_2_O, and 4 μL of DNA. The amplification was carried out in CFX Opus384 (BioRad) with the following protocol: initial denaturation (98 °C-30 s) followed by 40-amplification cycle program (98 °C-15 s and 65 °C-60 s). Normalized values were calculated with the CFX Manager program using *Rpl37a* as a reference gene.

### Immunostaining of rat seminiferous tubules

Testis samples were placed in OCT compound prior to cryostat sectioning and kept at − 80 °C until use. Samples were cut with a microtome set at 7 µm and mounted on Matsunami TOMO^®^ hydrophilic adhesion slides. Slides were left to dry at room temperature for about 30 min. Once dried, slides were fixed in 4% buffered paraformaldehyde for 8 min. Slides were quenched with 0.1 M Glycine in PBS and washed twice with PBS to remove residual glycine. A blocking solution (4% BSA in PBS) was added for 30 min at RT. Slides were then rinsed with PBS three times and rabbit anti-γH_2_AX (1:500, TREVIGEN, 4418-APC-100) was added diluted in DAKO. Antibodies were incubated overnight at 4 ºC. After rinsing, secondary antibodies also diluted in DAKO at a dilution of 1:500 (Invitrogen AlexaFluor^®^) were added and incubated at RT for 1 h. After three washes, Vectorshield solution containing DAPI was added to each slide. The cell count was performed using ImageJ. The ratio of γH2AX-positive cells divided by the total cell number in a seminiferous tubule was counted.

### Preparation and immunostaining of structurally preserved nuclei.

Structurally preserved nuclei (SPN) for three-dimensional analysis were prepared by cutting testis tissues in DMEM medium (Life Technologies, GIBCO) with 0.5% protease inhibitor. Testes were removed, and small piece of seminiferous tubules were placed in DMEM media. Structurally preserved suspension nuclei for imaging were prepared by mincing the seminiferous tissue with scalpels in cold DMEM containing Complete Mini EDTA-free protease inhibitor (Roche, 04693159001). 50 μL of cell suspension was mixed in equal volumes with equal amounts of 3.7% (vol/vol) paraformaldehyde and 0.1 M sucrose and spread on glass slides. The slides were air-dried and kept at − 80 °C. The slides were then washed several times with PBS and 2 min with 0.1 M glycine in PBS to remove the traces of paraformaldehyde before use. Cells were permeabilized by a 30 min incubation in PBS/0.5%Triton at RT, washed with PBS, and blocked for 30 min in a solution containing 0.1% (v/v) donkey serum, 0.03% (w/v) BSA, and 0.005% (v/v) Triton X-100 in PBS. SPN were immunostained with rabbit H3K9me3 (Abcam, ab8898, 1:500) and mouse monoclonal SYCP3 (1:200, Santa Cruz, SC-33195) antibodies at 4 °C overnight followed by several washes and incubation with Fluorescent Alexa antibodies. Z-stacks were acquired with 500 nm steps. 15 planes were taken for each individual channel, DAPI (Blue, 350 nm), SYCP3 (Red, 594 nm) and H3K9me3 (Green, 488 nm) using Zen Pro (version 2.3). All images for control and exposed samples were taken with fixed exposure times. Deconvolution was performed using the “Fast Iterative” algorithm provided by Zen Pro. The sum intensity images were generated for each z-stack and the resulting images were analyzed with ImageJ v1.52n. We used the lasso tool for nucleus contouring, and the integrated density immunofluorescence for each nucleus was calculated. The background area was subtracted from each image. We analyzed four independent biological replicates for control and treated groups. The data were plotted in Excel and presented as corrected total cell fluorescence (CTCF) of normalized fluorescence for nucleus ± SD, **p < 0.01, Mann-Whitney test.

### Hematoxylin–eosin staining

The slides for this procedure were prepared and analyzed in the same way as described for immunostaining. For morphology analysis, testes from control and nicotine-treated groups were fixed in 4% (w/v) PFA solution for 8 min and washed. Sections were stained with Hematoxylin and Eosin (H&E) according to standard protocol. Images were taken with NanoZoomer and quantitative analysis was performed using ImageJ. Images with tubules showing the presence of all cell types including the presence of elongated spermatids (stage VI or VII) were used for cell counts using ImageJ. Cells were counted and divided by the total area of the tubule. We counted cells in a minimum of 10 tubules using 4 biological replicates. Statistical significance was assessed with the Mann-Whitney test. For the seminiferous tubule epithelium and adluminal compartment height analysis, we measured the thickness from the bottom of the seminiferous tubule to the top of the cell layers. We measured diameters in (stage VI or VII) tubules found in each section. Statistical significance was assessed with a nonparametric Mann-Whitney test.

### Statistical analyses

We used the minimum number of animals required to obtain a statistically significant set of data according to the requirements of the USA ethics committee (IACUC). The number of animals used is specified for each experimental procedure. A nonparametric Wilcoxon-Mann-Whitney for RT-qPCR experiments, immunofluorescence, germ cells quantifications and WB assay was used.

### Supplementary Information

**Additional file 1.** Additional information; differentially methylated regions in the testis.

**Additional file 2.** Additional information; Differentially expressed genes in the pituitary gland.

**Additional file 3:** **Fig S1.** Uncut WB images.** Fig S2.** Ponceau red –stained membrane images. **Fig S3.** WB of PRM2. **Fig S4.** Examples of amplification and melting curves plots of methylation-specific PCR. Methylated and unmethylated PCR and melting plots are in shown in orange and green colors, respectively. **Fig S5.** The analysis of RNA-seq data in pituitary gland of rats. (A) Principal Component Analysis (PCA) plot, (B) a sample-to-sample dispersion heatmap, (C) MA-plot, (D) Volcano plot. **Table S1.** MeDIP sequencing reads at GRRs genes. **Table S2A.** The primers used for RT-qPCR in this study. **Table S2B.** The primers used in methylation-specific PCR. **Table S3.** The number of reads in MeDIP-seq libraries. **Table S4.** Number of reads in RNA-seq libraries.

## Data Availability

The datasets generated and analyzed during the current study are available in the NCBI Gene Expression Omnibus (GEO; https://www.ncbi.nlm.nih.gov/geo/) under the accession number GSE253596.

## Abbreviations

17-OHP: 17-Hydroxyprogesterone
Acrv1: Acrosomal vesicle protein 1
Acta2: Actin alpha 2, smooth muscle
Actc1: Actin, alpha, cardiac muscle 1
Adgrl3: Adhesion G protein-coupled receptor L3
ADHD: Attention-deficit/hyperactivity disorder
Adra2a: Adrenoceptor alpha 2A
Amhr2: Anti-Mullerian hormone receptor type 2
Anxa1: Annexin A1
Begain: Brain-enriched guanylate kinase-associated
Brdt: Bromodomain testis associated
Cabp1: Calcium binding protein 1
Col4a2: Collagen type IV alpha 2 chain
CrebB5: CAMP responsive element binding protein 5
Ctsl: Cathepsin L
Cyp1b1: Cytochrome P450, family 1, subfamily b, polypeptide 1
Dazl: Deleted in azoospermia-like
Ddx4: DEAD-box helicase 4
Dmd: Dystrophin
Dmtf1: Cyclin D binding myb-like transcription factor 1
Dnm2: Dynamin 2
Dtnbp1: Dystrobrevin binding protein 1
E2f7: E2F transcription factor 7
Efemp1: EGF containing fibulin extracellular matrix protein 1
Epha3: Eph receptor A3
Erbb4: Erb-b2 receptor tyrosine kinase 4
ETV4: ETS variant transcription factor 4
Foxj3: Forkhead box J3
Foxs1: Forkhead box S1
Gabra3: Gamma-aminobutyric acid type A receptor subunit alpha 3
Gabra4: Gamma-aminobutyric acid type A receptor subunit alpha 4
Gabrg2: Gamma-aminobutyric acid type A receptor subunit gamma 2
Gap43: Growth associated protein 43
GD: Gestational day
Gjb2: Gap junction protein, beta 2
Gpc6: Glypican 6
GRRs: Germ line reprogramming responsive genes
H2ax: H2A.X variant histone
Hormad1: HORMA domain containing 1
Hoxa2: Homeobox A2
Hoxa5: Homeobox protein Hox-A5
Hoxb3: Homeo box B3
Hoxb5: Homeo box B5
Hsf1: Heat shock transcription factor 1
Hsf5: Heat shock transcription factor 5
Igfbp6: Insulin-like growth factor binding protein 6
Igsf9b: Immunoglobulin superfamily member 9B
Iqsec2: IQ motif and Sec7 domain ArfGEF 2
Iqsec3: IQ motif and Sec7 domain ArfGEF 3
Kiss1: KiSS-1 metastasis-suppressor
Kit: KIT proto-oncogene receptor tyrosine kinase
Lgr4: Leucine-rich repeat-containing G protein-coupled receptor 4
MeDIP: Methylated DNA immunoprecipitation
Meis3: Meis homeobox 3
Mov10l1: Mov10 like RISC complex RNA helicase 1
Nfact2: Nuclear factor of activated T cells 2
Nrf1: Nuclear respiratory factor 1
Olfm1: Olfactomedin 1
Pgr: Progesterone receptor
Piwil1: Piwi-like RNA-mediated gene silencing 1
PNE: Prenatal nicotine exposure
Pnldc1: PARN like ribonuclease domain containing exonuclease 1
Prkar2a: Protein kinase cAMP-dependent type II regulatory subunit alpha
Prm1: Protamine 1
Prm2: Protamine 2
Psd2: Pleckstrin and Sec7 domain containing 2
Pttg1: PTTG1 regulator of sister chromatid separation, securin
Rad51: RAD51 recombinase
Rbpj: Recombination signal binding protein for immunoglobulin kappa J region
Rfx3: Regulatory factor X3
Slc32a1: Solute carrier family 32 member 1
Slc6a6: Solute carrier family 6 member 6
Slitrk1: SLIT and NTRK-like family, member 1
Slitrk2: SLIT and NTRK-like family, member 2
Spaca1: Sperm acrosome associated 1
Sst: Somatostatin
Stra8: Stimulated by retinoic acid 8
Syt10: Synaptotagmin 10
Tdrd1: Tudor domain containing 1
Tead1: TEA domain transcription factor 1
TET: Ten-eleven translocation
Tgfb1: Transforming growth factor, beta 1
Trpm2: Transient receptor potential cation channel, subfamily M, member 2
