# Supplementary material for: Prenatal nicotine exposure leads to epigenetic alterations in peripheral nervous system signaling genes in the testis of the rat
Source: Epigenetics Chromatin. 2024 May 7;17:14. doi: 10.1186/s13072-024-00539-5 (PMC11075221; doi:10.1186/s13072-024-00539-5)
Supplement: Supplementary file 3 — Additional file 3: Fig S1. Uncut WB images. Fig S2. Ponceau red –stained membrane images. Fig S3. WB of PRM2. Fig S4. Examples of amplification and melting curves plots of methylation-specific PCR. Methylated and unmethylated PCR and melting plots are in shown in orange and green colors, respectively. Fig S5. The analysis of RNA-seq data in pituitary gland of rats. (A) Principal Component Analysis (PCA) plot, (B) a sample-to-sample dispersion heatmap, (C) MA-plot, (D) Volcano plot. Table S1. MeDIP sequencing reads at GRRs genes. Table S2A. The primers used for RT-qPCR in this study. Table S2B. The primers used in methylation-specific PCR. Table S3. The number of reads in MeDIP-seq libraries. Table S4. Number of reads in RNA-seq libraries. [file 13072_2024_539_MOESM3_ESM.pdf]

Prenatal nicotine exposure leads to epigenetic alterations in peripheral nervous system signaling genes in the testis of the rat

Ouzna Dali[1]*, Jose Antonio Muriel Muriel[1]*, Ana Vargas Baco[1]*, Sergei Tevosian[2], Jasenka Zubcevic[3], Fatima Smagulova[1]#, Linda F. Hayward[2]

[1] Univ. Rennes, EHESP, Inserm, Irset (Institut de recherche en santé, environnement et travail) - UMR_S 1085, F-35000, Rennes, France

[2] University of Florida, Department of Physiological Sciences Box 100144, 1333 Center Drive, 32610, Gainesville, FL, USA

[3] University of Toledo, Department of Physiology and Pharmacology, Toledo, Ohio, USA

Supplementary information

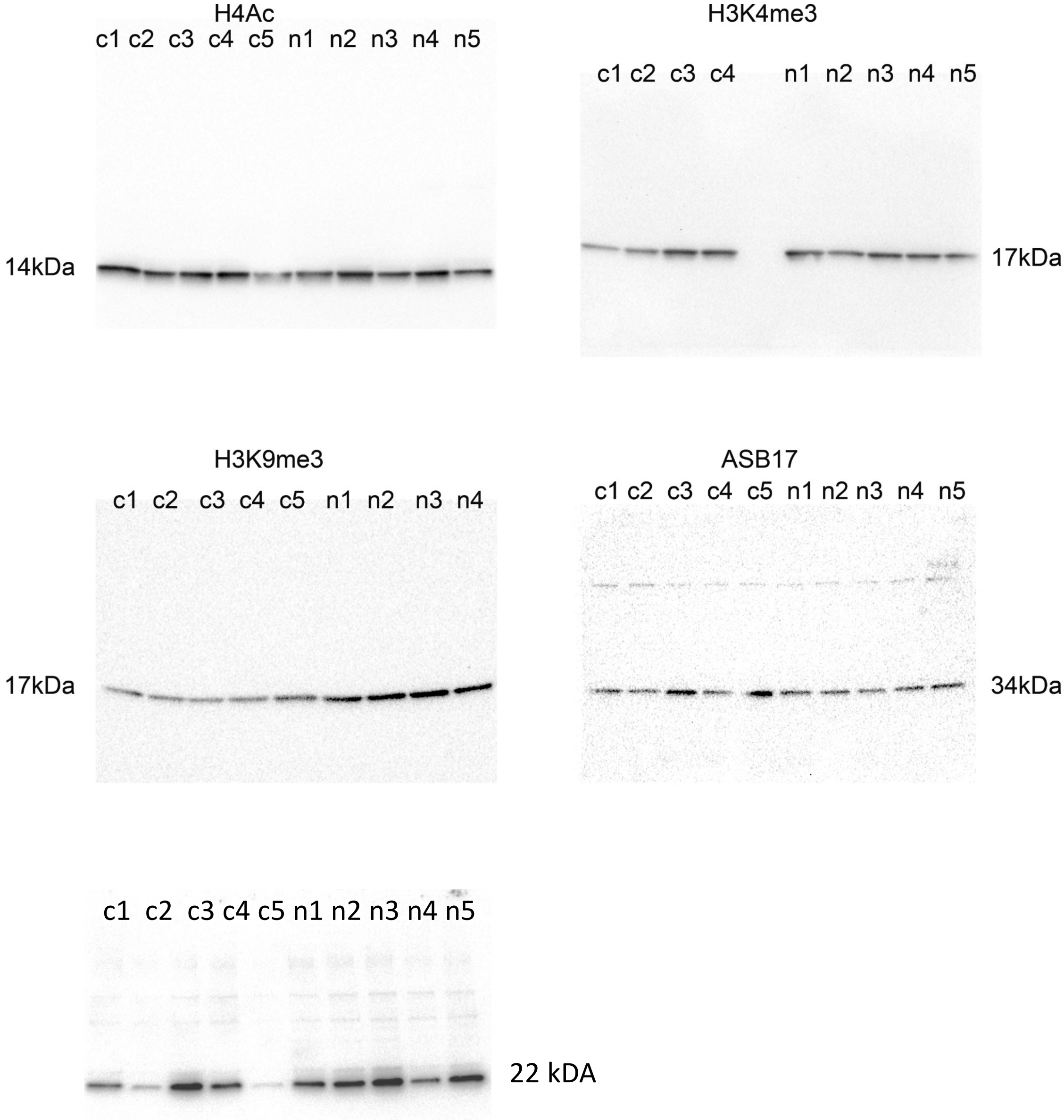

Fig S1. Uncut WB images

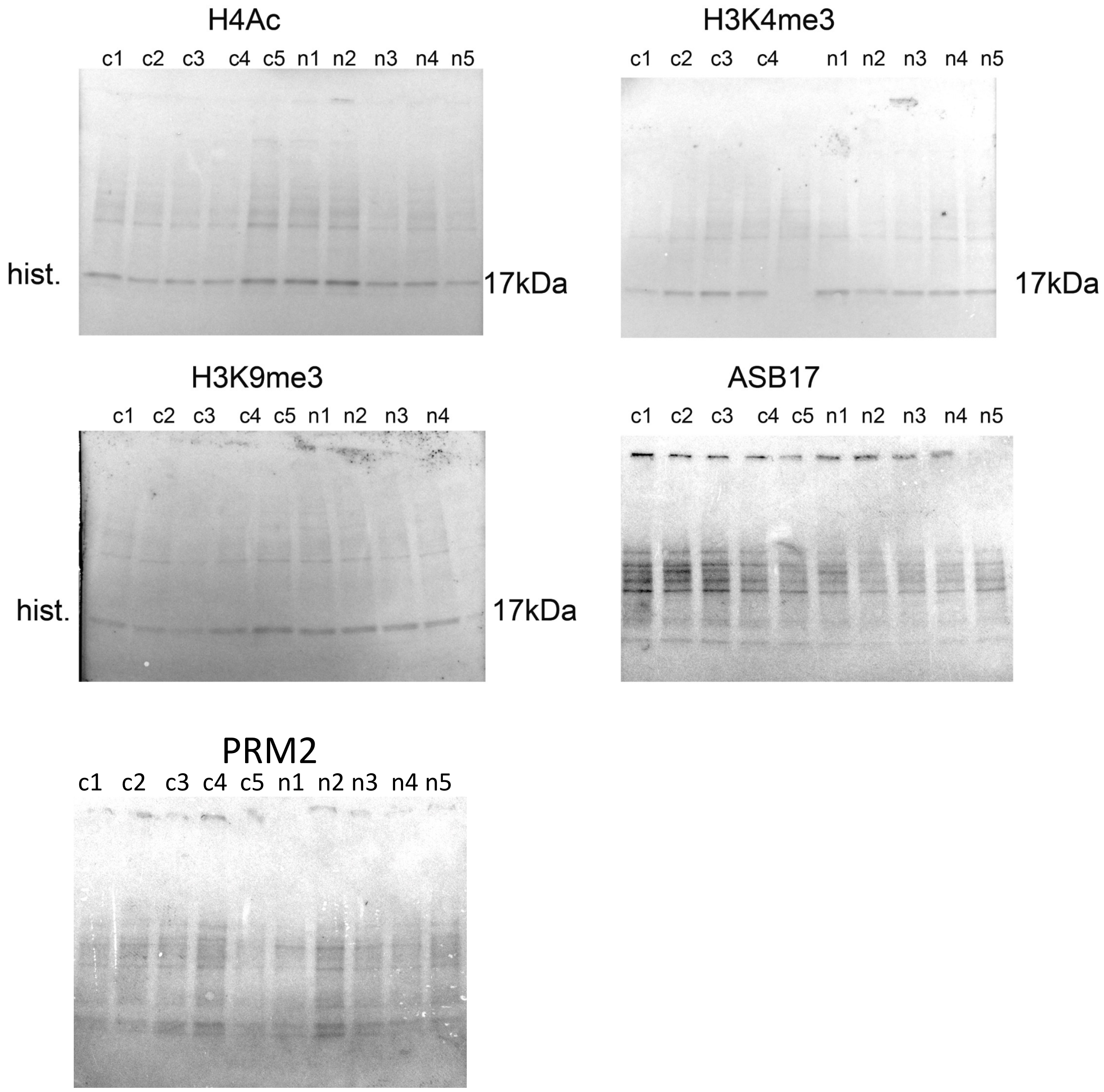

Fig S2. Ponceau red –stained membrane images

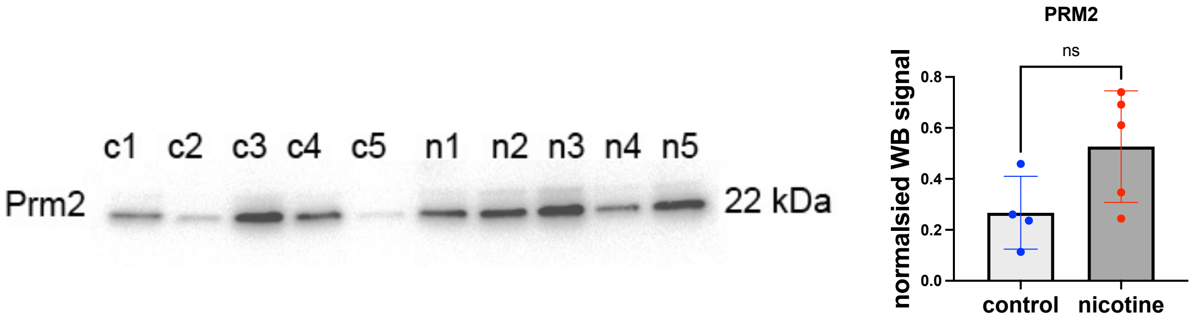

Fig S3. WB of PRM2

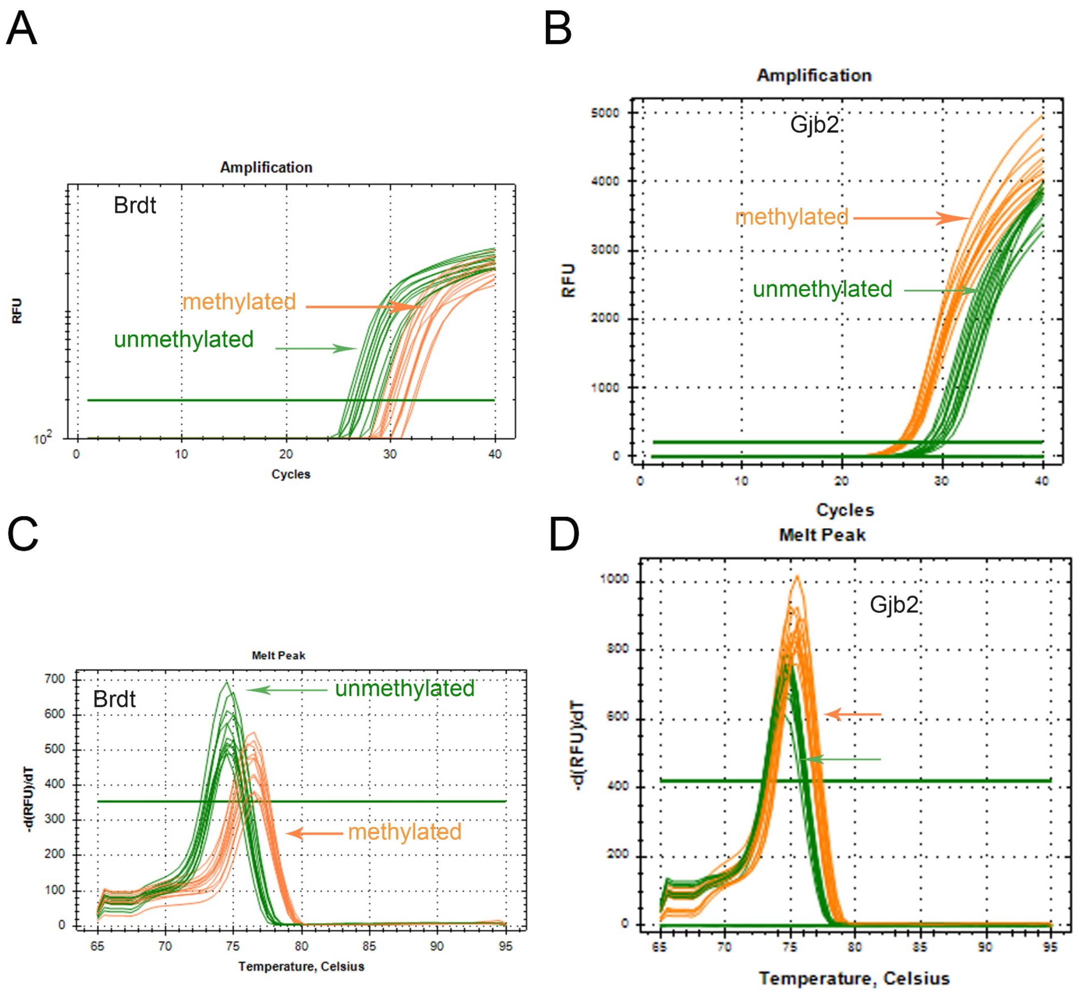

Fig S4. Examples of amplification and melting curves plots of methylation-specific PCR. Methylated and unmethylated PCR and melting plots are in shown in orange and green colors, respectively.

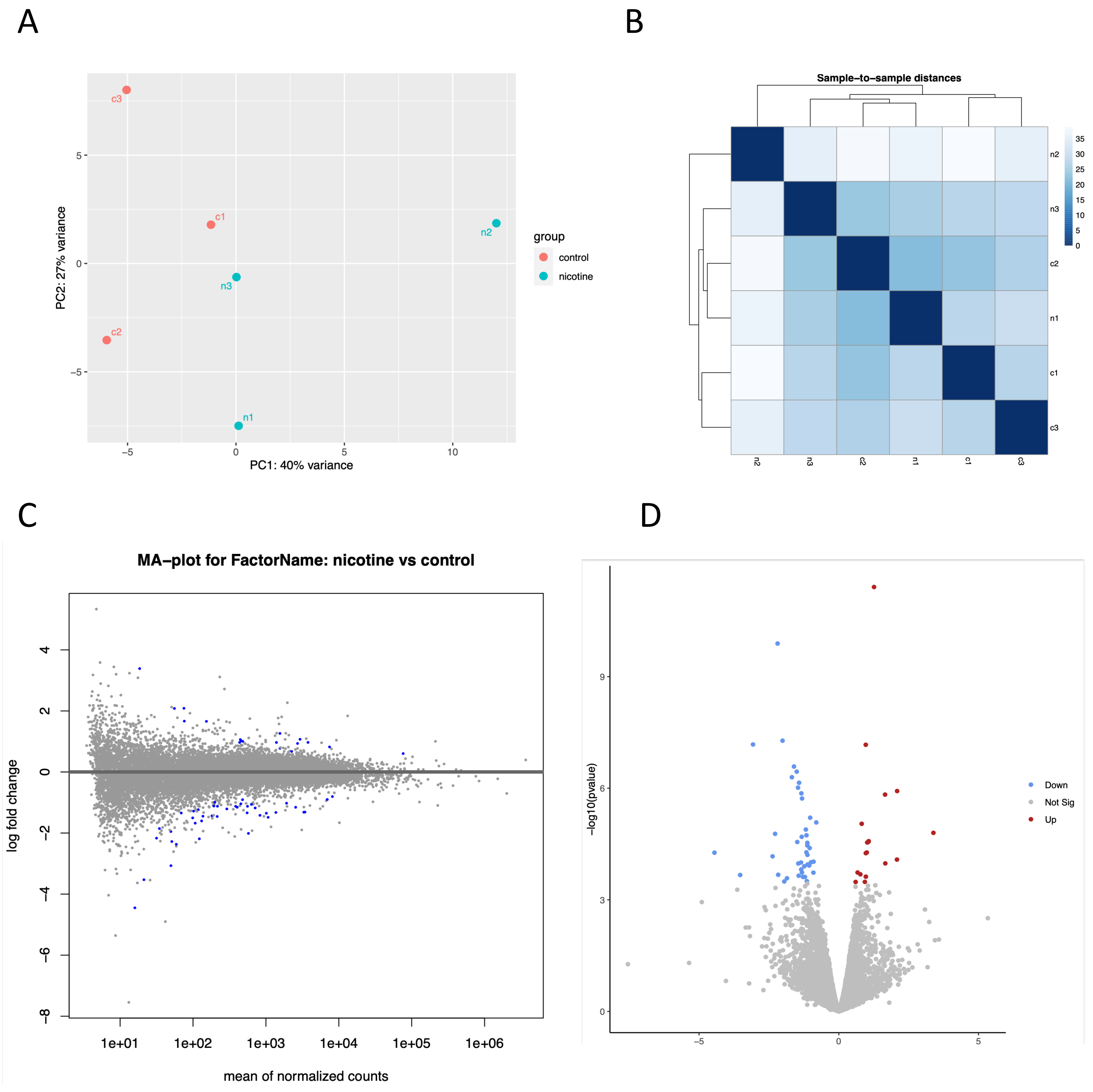

Fig S5. The analysis of RNA-seq data in pituitary gland of rats. (A) Principal Component Analysis (PCA) plot, (B) a sample-to-sample dispersion heatmap, (C) MA-plot, (D) Volcano plot

Table S1. MeDIP sequencing reads at GRRs genes

GRRS	coordinates	c1	c2	c3	n1	n2	n3
Adad1	chr2:119948550-119949362	948	742	467	379	488	620
Asz1	chr4:46455672-46456268	72	46	68	54	49	90
Brdt	chr14:2445279-2446111	306	305	263	213	287	190
Dazl	chr9:10694834-10696526	1729	1430	951	1032	1305	1174
Ddx4	chr2:44282447-44283190	956	856	517	508	755	512
Dpep3	chr19:33872289-33872677	16	11	23	12	2	11
Fam178b	chr9:38913480-38914080	232	242	172	169	220	165
Fkbp6	chr12:21319205-21320021	745	534	493	410	427	423
Hormad1	chr2:183116388-183117077	130	141	97	111	105	136
Hsf2bp	chr20:10120996-10121655	402	286	305	369	256	231
Hsf5	chr10:72416717-72418109	922	691	577	501	477	434
Mael	chr13:78496095-78496994	867	717	565	463	666	570
Mov10l1	chr7:120087902-120088677	321	453	366	570	687	627
Naa11	chr14:12183322-12184316	1007	901	697	688	735	672
Pnldc1	chr1:47842878-47843597	536	395	357	266	285	286
Rad51c	chr10:72231567-72232071	78	100	91	62	39	67
Rhox13	chrX:116910978-116912062	229	239	207	273	194	216
Rpl10l	chr6:84545037-84546118	729	787	557	619	752	492
Sec1	chr1:96164804-96165771	1228	955	617	655	894	726
Slc25a31	chr2:123695115-123695948	550	423	495	341	349	411
Stk31	chr4:78370450-78371220	704	525	438	402	509	383
Sycp1	chr2:190456144-190456930	582	372	307	333	343	392
Sycp2	chr3:165498312-165499122	590	383	336	308	343	408
Sycp3	chr7:22873818-22874669	295	260	232	290	277	266
Taf7l	chrX:97672901-97674576	402	222	199	196	201	191
Taf9b	chrX:71299746-71300896	914	873	563	497	577	489
Tdrd1	chr1:255875452-255876333	1192	838	630	593	673	595
Tex12	chr8:50912802-50913478	625	407	361	341	346	347
Trim52	chr15:82885997-82886967	1015	723	636	643	712	594

Table S2A. The primers used for RT-qPCR in this study

Gene	Forward (+)	Reverse	
Brdt	CGAAGCTCAAGCTGCCTGACTATT	CTCCCACGCATTCGGAAGCC	GRR
Dazl	TGGTGTGTCGAAGGGCTATGG	CCTGATTGCAGGGCCCAGTT	GRR
Ddx4	AGCAGCAAGTGATTCAGGCAGT	ACTGCTTTCGCCTCCTTCAGC	GRR
Hormad1	CGGAGGACAGCTTCTATGAGTGC	GGATACTGAAACAGCCAGGAGCC	GRR
Sycp1	GCCCATGCTTGAACAGGTTGC	TGAGTACAGTCTGCTCATTGGCT	GRR
Tdrd1	CTTTTTCTGGGTCAGGCGGC	CTTAACATTCACTGACGTCTCAACG	GRR
Sycp1	GCCCATGCTTGAACAGGTTGC	TGAGTACAGTCTGCTCATTGGCT	GRR
Rpl37a	TGGGGCCTGGACCTACAATACC	ACCAGGCAAGTCTCAGGAGGT	housekeep.
H2afx	CCCTTTTAAGGGCCACCACCT	GGGAAGGGTAAAGACGCGAGG	SC
Mybl1	CGATACTGGGCACCACACCA	GGCAAGTGGCTGGGACACAA	SC
Piwil1	GCACGGGGCCAAGATAGGAC	GGCTCGTCCAGTCATTTTCCAG	SC
Pttg1	GGCGTAGTCTTGGGTCTCTCC	GACACCAGAGCCCAGCTTCAA	SC
Rad51	CCACGGCTGCGAGACAGTAAT	ACCGAGAAATAGGCTGTGGACC	SC
Amhr2	TCTTCCAAGGAAGCGTTGACGA	GGTCCTCCTGTTTGGGGATACTTG	Sertoli
Ctsl	CGCCTTCGGTGACATGACCA	GGGGATCTGCAGCATCAGAGG	Sertoli
Kiss1	GATCTCGCTGGCTTCTTGGCAG	TCCGGACTGTTGGCCTGTGG	Sertoli
Kit	CAACGGCACGGTGGAGTGTA	GGGGCTGGATTTGCTCTTTGC	SG
Lgr4	CTCCGGAAAGGGGTTGACGG	ACCAGCCAGTTGTAGCTCCTC	SG
Stra8	ACAGCCTCAAAGTGGCAGGT	TGGGATTTCCGTCTTGCAGGT	SG
Acrv1	CAAGCACGTCTTCAGGTGGGA	TGCTGGGAATTTTGAGTGGTGC	ST
Prm1	CAGCCCACAAAATTCCACCTGC	TCTGGCCATGGTGCTAGCTT	ST
Spaca1	CCAGACCAGACACTGATGCCG	AGGATTTCACTGCCGCCCAAT	ST
Prm2	GACAGAAAGGGGCCACCACC	TCGGGATCTTCTGCAGCCTCT	ST

Table S2B. The primers used in methylation-specific PCR

chr9_Dazl_BS_m	TTCGCGTATGTTTTATGCGT	GAAAATAACTATACGTAACTAACCTCGTA	testis
chr9_Dazl_BS_um	TTGTATTTTGTGTATGTTTTATGTGT	AAAAATAACTATACATAACTAACCTCATA	testis
chr1_Pnldc1_BS_m	TTAGGTTTTGGTTTTGGTGTAATAC	ATAAAAAACTCTACTCAAATTCGTC	testis
chr1_Pnldc1_BS_um	GGTTTTGGTTTTGGTGTAATATGT	AAATAAAAAACTCTACTCAAATTCATC	testis
chr1_Tdrd1_BS_m	GTTGCGGGGTATGGTAAATATC	ACTCTAAACTTCCGACTCCTAACG	testis
chr1_Tdrd1_BS_um	GGTTGTGGGGTATGGTAAATATT	CAACTCTAAACTTCCAACTCCTAACA	testis
chr10_Hsf5_BS_m	GGTGTTTTGTCGTTCGTTTAATC	ATATTCATTCCCTACTACCCACGTA	testis
chr10_Hsf5_BS_um	GAGGTGTTTTGTTGTTTGTTTAATT	ATATTCATTCCCTACTACCCACATA	testis
chr14_Brdt_BS_m	TCGATATTTTAATTACGAAGGAATC	TTACCCAACCGAATACGTACG	testis
chr14_Brdt_BS_um	TTTGATATTTTAATTATGAAGGAATTGT	CATTACCCAACCAAATACATACAAC	testis
chr14_Efemp1_Bs_m	GATTCGGTGAGAGAGAGATTTAATC	GATATCTCAAACAAAAATCCTACCG	brain
chr14_Efemp1_Bs_um	TTTGGTGAGAGAGAGATTTAATTGT	CAATATCTCAAACAAAAATCCTACCA	brain
chr15_Gjb2_BS_m	TTTATATTTTTTGGGCGGTTATTAC	AAAAACCCTACACACTATCATCGAC	brain
chr15_Gjb2_BS_um	TATATTTTTTGGGTGGTTATTATGA	AAAAACCCTACACACTATCATCAAC	brain
chr2_Ddx4_BS_m	GTTATCGGGTTTGGTTAGGAC	CGTCGCCTAATACTATTTATTATCGA	testis
chr2_Ddx4_BS_um	GTTATTGGGTTTGGTTAGGATGT	CCCCATCACCTAATACTATTTATTATCA	testis
chr7_Igfbp6_BS_m	TGTAGTGTTAATTTCGAGAGAACGA	CCTCAAATAAACGAATAAAAAACGA	brain
chr7_Igfbp6_BS_um	TGTAGTGTTAATTTTGAGAGAATGA	CTCAAATAAACAAATAAAAAACAAA	brain
chr9_Erbb4_BS_m	TATTATTATGTTTGTGGTCGAGGTC	GCTTAACCGATACTACCCTACGTA	brain
chr9_Erbb4_BS_um	TTATTATGTTTGTGGTTGAGGTTGT	CATCACTTAACCAATACTACCCTACATA	brain

Table S3. The number of reads in MeDIP-seq libraries

sample	read number	group
FMSV483	4,16E+07	control
FMSV484	2,66E+07	control
FMSV485	3,77E+07	control
FMSV486	3,66E+07	nicotine
FMSV487	2,65E+07	nicotine
FMSV488	3,00E+07	nicotine
FMSV497	1,09E+08	input

Table S4. Number of reads in RNA-seq libraries

sample	read number	group
FMSV439	3,40E+07	nicotine
FMSV440	6,49E+07	nicotine
FMSV441	4,40E+07	nicotine
FMSV442	5,37E+07	control
FMSV443	9,73E+07	control
FMSV444	7,31E+07	control
